# From Repurposing to Redesign: Optimization of Boceprevir to Highly Potent Inhibitors of the SARS-CoV-2 Main Protease †

**DOI:** 10.3390/molecules27134292

**Published:** 2022-07-04

**Authors:** Matthias Göhl, Linlin Zhang, Haifa El Kilani, Xinyuanyuan Sun, Kaixuan Zhang, Mark Brönstrup, Rolf Hilgenfeld

**Affiliations:** 1 Department of Chemical Biology, Helmholtz Centre for Infection Research, Inhoffenstr. 7, 38124 Braunschweig, Germany; matthias.goehl@helmholtz-hzi.de; 2 Institute of Molecular Medicine, University of Lübeck, Ratzeburger Allee 160, 23562 Lübeck, Germany; llzhang@biochem.uni-luebeck.de (L.Z.); haifa.elkilani@uni-luebeck.de (H.E.K.); xinyuanyuan.sun@uni-luebeck.de (X.S.); kaixuan.zhang@uni-luebeck.de (K.Z.); 3 German Center for Infection Research (DZIF), Hannover-Braunschweig Site, 38124 Braunschweig, Germany; 4 German Center for Infection Research (DZIF), Hamburg-Lübeck-Borstel-Riems Site, 23562 Lübeck, Germany

**Keywords:** SARS-CoV-2, COVID-19, main protease, 3C-like protease, enterovirus 3C protease, Coxsackievirus B3, structure-based drug design, alpha-ketoamides, boceprevir, telaprevir, X-ray crystallography

## Abstract

The main protease (M^pro^) of the betacoronavirus SARS-CoV-2 is an attractive target for the development of treatments for COVID-19. Structure-based design is a successful approach to discovering new inhibitors of the M^pro^. Starting from crystal structures of the M^pro^ in complexes with the Hepatitis C virus NS3/4A protease inhibitors boceprevir and telaprevir, we optimized the potency of the alpha-ketoamide boceprevir against the M^pro^ by replacing its P1 cyclobutyl moiety by a γ-lactam as a glutamine surrogate. The resulting compound, **MG-78**, exhibited an IC_50_ of 13 nM versus the recombinant M^pro^, and similar potency was observed for its P1′ *N*-methyl derivative **MG-131**. Crystal structures confirmed the validity of our design concept. In addition to SARS-CoV-2 M^pro^ inhibition, we also explored the activity of **MG-78** against the M^pro^ of the alphacoronavirus HCoV NL63 and against enterovirus 3C proteases. The activities were good (0.33 µM, HCoV-NL63 M^pro^), moderate (1.45 µM, Coxsackievirus 3C^pro^), and relatively poor (6.7 µM, enterovirus A71 3C^pro^), respectively. The structural basis for the differences in activities was revealed by X-ray crystallo-graphy. We conclude that the modified boceprevir scaffold is suitable for obtaining high-potency inhibitors of the coronavirus M^pro^s but further optimization would be needed to target enterovirus 3C^pro^s efficiently.

## 1. Introduction

The main protease (M^pro^) of SARS-CoV-2 is a prime target for antiviral drug discovery and development [1,2,3,4,5,6,7,8]. Previously, we have described peptidomimetic α-ketoamides as nearly equipotent inhibitors of the M^pro^ of betacoronaviruses (such as SARS-CoV and MERS-CoV) as well as the 3C^pro^ of enteroviruses [9]. Interestingly, we found it more challenging to achieve equipotency against M^pro^s of alphacoronaviruses such as HCoV NL63. The most potent inhibitor in this series was **11r**, with an IC_50_ of 0.71 μM against the SARS-CoV M^pro^ (0.87 μM against the SARS-CoV-2 orthologue), 0.95 μM against the Coxsackievirus B3 (CVB3) 3C^pro^, and 1.69 μM against the enterovirus A71 (EV-A71) 3C^pro^ [9]. In a second series of compounds, we optimized the pharmacokinetic properties of these inhibitors. Meanwhile, the COVID-19 pandemic had arrived and with it a shift of priorities—there was no need any longer to find compromises between the steric demands of coronavirus M^pro^s and enterovirus 3C^pro^s. Early in 2020, we determined the crystal structure of the SARS-CoV-2 M^pro^ and used it as a basis to design compound **13b**, with IC_50_ = 0.67 μM against the target enzyme [10]. Since then, we have optimized our enzyme assay; under the improved conditions, compound **13b** now exhibits IC_50_ = 0.38 μM. Following further optimization to yield **13b-K** (Cooper et al., to be submitted), the compound is now entering efficacy trials in a small-animal model.

**Figure 1:**
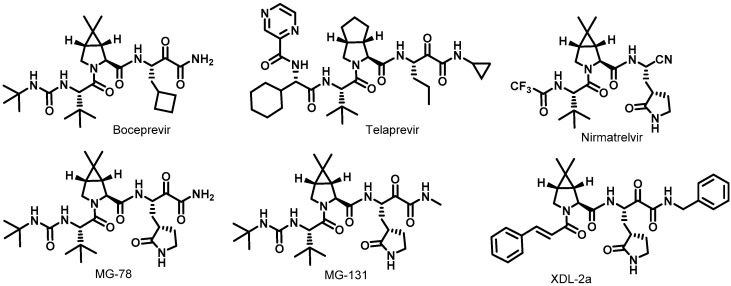
Chemical structures of viral protease inhibitors.

Here we report another two α-ketoamides, compounds **MG-78** and **MG-131**, which we designed based on the crystal structure of the SARS-CoV-2 M^pro^ in complex with boceprevir, an inhibitor of the hepatitis C virus NS3/4A protease (Figure 1). We determined the crystal structure of this latter complex as well as that of the main protease with the related drug telaprevir in June, 2020. However, at that time, several crystallographic studies of the two complexes were published as part of drug repurposing studies for the treatment of COVID-19 (for boceprevir, see [11,12,13,14], with PDB codes 6ZRU, 7BRP, 7COM, 6XQU; for telaprevir, see [11,13,14], with PDB codes 6ZRT, 7C7P, and 6XQS), so that we only deposited the atomic coordinates and structure factors in the Protein Data Bank (PDB codes 7NBR and 7NBS). Hence, we only mention some essential aspects of these crystal structures in the Section 2 “Results” below.

Boceprevir and telaprevir show moderate and weak inhibition, respectively, against the SARS-CoV-2 M^pro^. The crystal structures reveal an overall acceptable fit between the drugs and the M^pro^ but also less-than-ideal interactions and, in the case of telaprevir, costly conformational changes that are likely responsible for the reduced inhibitory potency. For both compounds, the P1 moiety (cyclobutyl methyl for boceprevir, and *n*-propyl for telaprevir) does not constitute a good fit to the S1 specificity site of the M^pro^. We therefore replaced it by the canonical γ-lactam typical for coronavirus M^pro^ inhibitors at the P1 position, yielding the α-ketoamide **MG-78**. In addition, we prepared **MG-131**, the P1′-*N*-methyl derivative of **MG-78** (Figure 1). The synthesis and biochemical characterization of these compounds, as well as the crystal structures of their complexes with the SARS-CoV-2 M^pro^, are reported below.

In line with our original goal of identifying broad-spectrum inhibitors that target the main protease of coronaviruses and the 3C protease of enteroviruses, we also assessed the inhibitory potency of **MG-78** against the M^pro^ of the alphacoronavirus HCoV NL63 and the 3C^pro^s of enterovirus A71 (EV-A71) and Coxsackievirus B3 (CVB3). For the latter two, we also present crystal structures of their complexes with **MG-78**, explaining the observed activities.

## 2. Results

### 2.1. Inhibition of the SARS-CoV-2 Main Protease by Boceprevir and Telaprevir

The inhibitory potency of boceprevir and telaprevir against the recombinant SARS-CoV-2 M^pro^ was assessed by using an established Förster resonance energy transfer (FRET) assay with the M^pro^ substrate Dabcyl-KTSAVLQ↓SGFRKM-E(Edans)-NH_2_ (↓ indicates the protease cleavage site). Boceprevir was found to inhibit the cleavage reaction with an IC_50_ of 4.1 ± 0.9 μM, whereas this value was 19.0 ± 9.8 μM for telaprevir (Figure S1). Thus, these compounds, in particular telaprevir, are considerably weaker inhibitors of the SARS-CoV-2 main protease compared to our lead compound **13b** [10] (IC_50_ = 0.38 μM for the mixture of active and inactive diastereomers).

### 2.2. Crystal Structures of SARS-CoV-2 M^pro^ Complexes with Boceprevir and Telaprevir

Boceprevir and telaprevir are peptidomimetic α-ketoamides carrying a P2-bicyclic proline derivative as their hallmark. The sulfur atom of the nucleophilic Cys145 of the M^pro^ is covalently linked to the α-carbon of the inhibitor’s ketoamide warhead. As the binding affinity of these compounds is mainly controlled by the P1′, P1, and P2 moieties interacting with the target protease, we restrict our structural description to these.

### 2.3. P1′ Moiety

Boceprevir has no substituent on the amide nitrogen of the warhead, whereas telaprevir has a cyclopropyl residue attached to the nitrogen atom. The cyclopropyl substituent of telaprevir fills the S1′ pocket of the M^pro^, with hydrophobic contacts to Thr25, Thr26, and Asn142. Actually, the contact to the carbonyl oxygen of Thr26 is somewhat short, possibly contributing to the poor inhibitory potency of telaprevir towards the SARS-CoV-2 M^pro^. Compound **13b** has a larger benzyl substituent in this position, but its phenyl ring is located outside the S1′ pocket [10]. A higher affinity might be achieved through smaller substituents at this position, e.g., by an *N*-methyl group (as in compound **MG-131**, see below).

### 2.4. P1 Substituent

Boceprevir and telaprevir have a cyclobutylmethyl and *n*-propyl moiety, respectively, in the P1 position. These substituents are too short and too hydrophobic to interact with His163 (5.6/3.8 Å away in the boceprevir/telaprevir complexes), the Glu166 carboxylate (4.6/5.1 Å away), and the main-chain oxygen of Phe140 (4.4/3.7 Å away), the anchoring points for the P1-lactam moiety of M^pro^ inhibitors near the bottom of the enzyme’s S1 pocket. Consequently, it can be assumed that the P1 substituents of boceprevir and telaprevir contribute little to the binding affinity of the compounds towards the SARS-CoV-2 M^pro^.

### 2.5. P2 Position

Boceprevir comprises a *gem*-dimethyl cyclopropane ring annealed to a proline as P2 moiety, whereas in telaprevir, the dimethyl cyclopropane is replaced by cyclopentane. The bicyclic system fits into the S2 pocket of the M^pro^, which has a volume of 216.5/257.2 Å^3^ in the boceprevir/telaprevir complexes, respectively, as calculated using UCSF-Chimera [15]. We have previously emphasized the plasticity of the S2 pocket of the betacoronavirus M^pro^s, which, mainly due to the flexibility of the side-chains of Gln189, Met49, and Met165, can adapt to the size of the P2 substituent and accommodate small moieties such as cyclopropylmethyl (as in compounds **11q** and **13b** [9,10]) or larger ones such as cyclopentylmethyl or cyclohexylmethyl (as in compounds **11u** and **11r** or **13a**, respectively [9,10]), with a preference for smaller ones.

Because of the tetrahedral arrangement of the two methyl substituents at the tip of the cyclopropane part of boceprevir’s bicyclic P2 moiety, they are almost orthogonal to the plane of the cyclopropane and fit to the sterically demanding S2 pocket of the main protease. In contrast, telaprevir has a cyclopentane ring fused to the proline. This nearly planar cycloalkane leads to contacts with polar atoms of residues Asp187 or Arg188 at the back-wall of the pocket, in addition to hydrophobic contacts to Met165 at the bottom of the S2 site and His41 of its side-wall. The cycloalkane rings fused to the proline describe angles of ~120° and ~110° with the proline in boceprevir and telaprevir, respectively. In boceprevir, this angle is the same in the complexes with the HCV NS3/4A protease [16] (PDB code: 2CO8) and the SARS-CoV-2 M^pro^, whereas in telaprevir [17] (PDB code: 3SV6), the angle changes from ~117° (in the HCV NS3/4A complex) to ~110° (in the M^pro^ complex). In addition, there are slight changes in the ring pucker of both the proline and the cyclopentane in telaprevir, as revealed by a comparison of the torsion angles with the structure of the compound in the complex with the HCV NS3/4A protease. The reason for the changes in telaprevir, which are certainly costly in enthalpic terms, is that in the absence of such “tilting” (decrease in the angle between the two rings and change of ring pucker), the tip of the cyclopentane would probably be too close to the Cβ atom of Asp187. Furthermore, the distance from the cyclopentane Cδ2 to Met49 Cε would be only 2.5 Å and that from Cδ2 to Met165 Cβ 2.9 Å. In the tilted orientation, these distances are 3.5 and 4.3 Å, respectively. In the HCV protease complex [17], stacking between the cyclopentane and the His56 imidazole is observed. By contrast, in the SARS-CoV-2 M^pro^ complex, there might be a C-H···π-H-bond [18,19] to His41.

### 2.6. Design and Synthesis of ***MG-78***

Whereas the inhibitory potency of boceprevir and telaprevir towards the M^pro^ appeared insufficient for a direct repurposing of the drugs against SARS-CoV-2, both compounds bring clear advantages such as oral bioavailability and a favorable toxicity profile. We therefore aimed to optimize their enzymatic activity against M^pro^, but keep structural changes to a minimum at the same time in order to retain their benefits. Because of its higher potency, we selected the boceprevir scaffold, and replaced its P1-cyclobutyl moiety with the γ-lactam residue that was found to be an optimal residue for the corresponding S1 pocket of M^pro^ in previous studies [20,21] (see also [9,10]), to yield **MG-78** (Figure 1). The key disconnection between the γ-lactam building block **4_a_** and the cyclopropyl proline residue was adapted from optimized synthetic procedures reported for boceprevir [22].

**Scheme 1:**
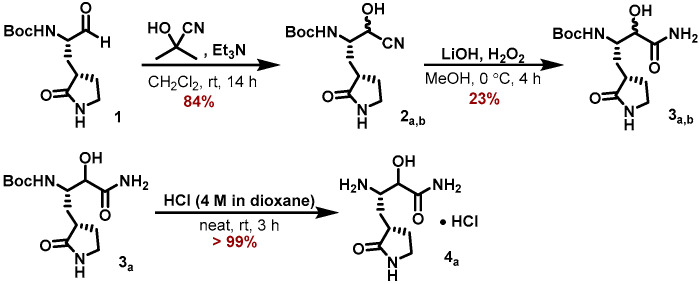
Synthesis of the γ-lactam building block **4_a_**.

The synthetic sequence for the γ-lactam building block **4_a_** is outlined in Scheme 1 and starts with the known aldehyde **1** [22]). Using acetone cyanohydrin as a HCN source in the presence of Et_3_N converted aldehyde **1** to a mixture of the corresponding cyanohydrin diastereomers **2_a,b_**. Hydrolysis of the cyanide group was accomplished by in situ-generated LiOOH in MeOH, and the corresponding α-hydroxy-amides **3_a,b_** were separated as part of the necessary purification by RP-18 HPLC. The major diastereomer **3_a_** was deprotected with HCl in 1,4-dioxane to furnish the corresponding ammonium chloride **4_a_**.

**Scheme 2:**
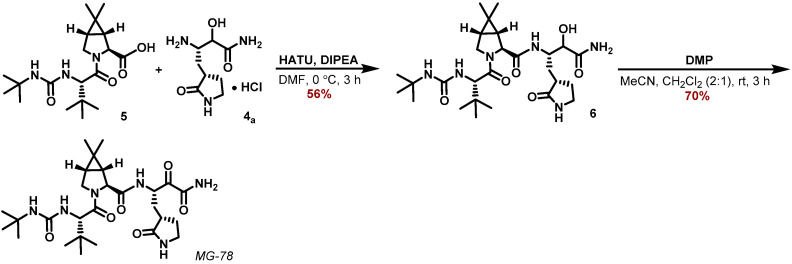
Peptide coupling of **5** and **4_a_** to the amide **6,** followed by DMP oxidation to the ketoamide **MG-78**.

Peptide coupling of the known carboxylic acid **5 [22]** with ammonium chloride **4_a_** applying HATU and DIPEA as a base in DMF at 0 °C furnished the α-hydroxy amide **6** (Scheme 2). Finally, DMP oxidation in a MeCN/CH_2_Cl_2_ mixture provided the α-ketoamide **MG-78**.

**Scheme 3:**
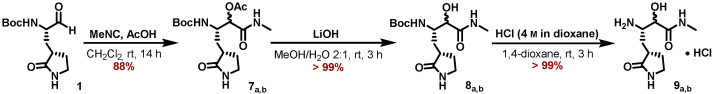
Synthesis of the γ-lactam building block **9_a,b_**.

A similar approach was applied for the synthesis of the N-methyl ketoamide derivative **MG-131**. Passerini reaction of the aldehyde **1** with isocyanomethane and AcOH in CH_2_Cl_2_ furnished the α-acetoxy amides **7_a,b_** as an inconsequential mixture of diastereomers in 88% yield (Scheme 3). Saponification of the acetoxy moiety with LiOH in a MeOH/H_2_O mixture provided the α-hydroxy amides **8_a,b_**, which were deprotected with HCl in 1,4-dioxane to deliver the corresponding ammonium salts **9_a,b_**.

**Scheme 4:**
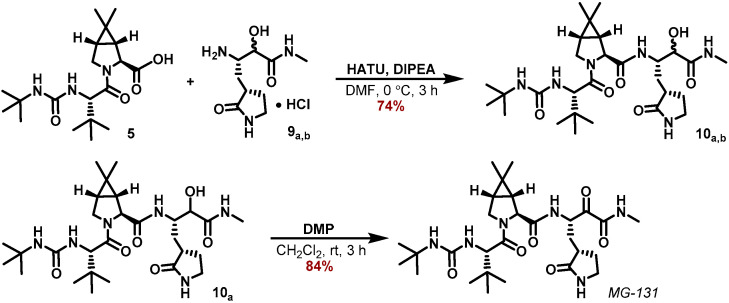
Peptide coupling of the carboxylic acid **5** and the ammonium salts **9_a,b_** to the amides **10_a,b_**, followed by DMP oxidation of the major diastereomer **10_a_** to the ketoamide **MG-131**.

Subsequent HATU coupling with the carboxylic acid **5** using DIPEA as base in DMF at 0 °C, provided the amides **10_a,b_**. Those were finally purified and separated by RP-18 HPLC in 74% yield for the sum of both diastereomers. DMP-oxidation of the major diastereomer **10_a_** in CH_2_Cl_2_ furnished the ketoamide **MG-131** in 84% yield (Scheme 4).

Overall, the ketoamides **MG-78** and **MG-131** could be synthesized from the aldehyde **1** in five steps each. Although the hydrolysis of the cyanohydrins **2_a,b_** to the α-hydroxy-amides **3_a,b_** can still be optimized (Scheme 1), the outlined approach provides a robust synthetic route to the ketoamide **MG-78** in 8% overall yield. The corresponding N-methyl ketoamide **MG-131** could be prepared in a similar reaction sequence in 55% overall yield.

**MG-78** was then tested for its inhibitory activity against the SARS-CoV-2 M^pro^. To our delight, the compound turned out to be highly potent with an IC_50_ of 13 ± 3 nM (Figure S1). The inhibitory activity of **MG-131** against the SARS-CoV-2 M^pro^ was found to be equal to that of **MG-78**, with IC_50_ = 14 ± 3 nM.

The pronounced increase in potency compared to boceprevir provides strong evidence for our design rationale being correct. To further substantiate this, we analyzed the cocrystal structures of the SARS-CoV-2 M^pro^ with either compound.

### 2.7. Crystal Structures of the SARS-CoV M^pro^ Complexes with ***MG-78*** and ***MG-131***

The complex between the M^pro^ and **MG-78** crystallized in space group P2_1_2_1_2, whereas the complex with **MG-131** featured space group C2. Both structures contain half an M^pro^ homodimer per asymmetric unit. The resolution of the structures is 1.8 and 2.5 Å, respectively, for the **MG-78** and the **MG-131** complex. The r.m.s. distance between all atoms of the two structures is 1.1 Å (0.4 Å for 252 well-aligned Cα atom pairs (out of 304 common Cαs)). Structural differences between the two molecules are restricted to amino-acid residues located at the surface of the protein. For example, the side-chain of His64 adopts two different orientations due to different crystal contacts. Apart from these minor differences, the two structures are highly similar and will hence be discussed together here.

**Figure 2:**
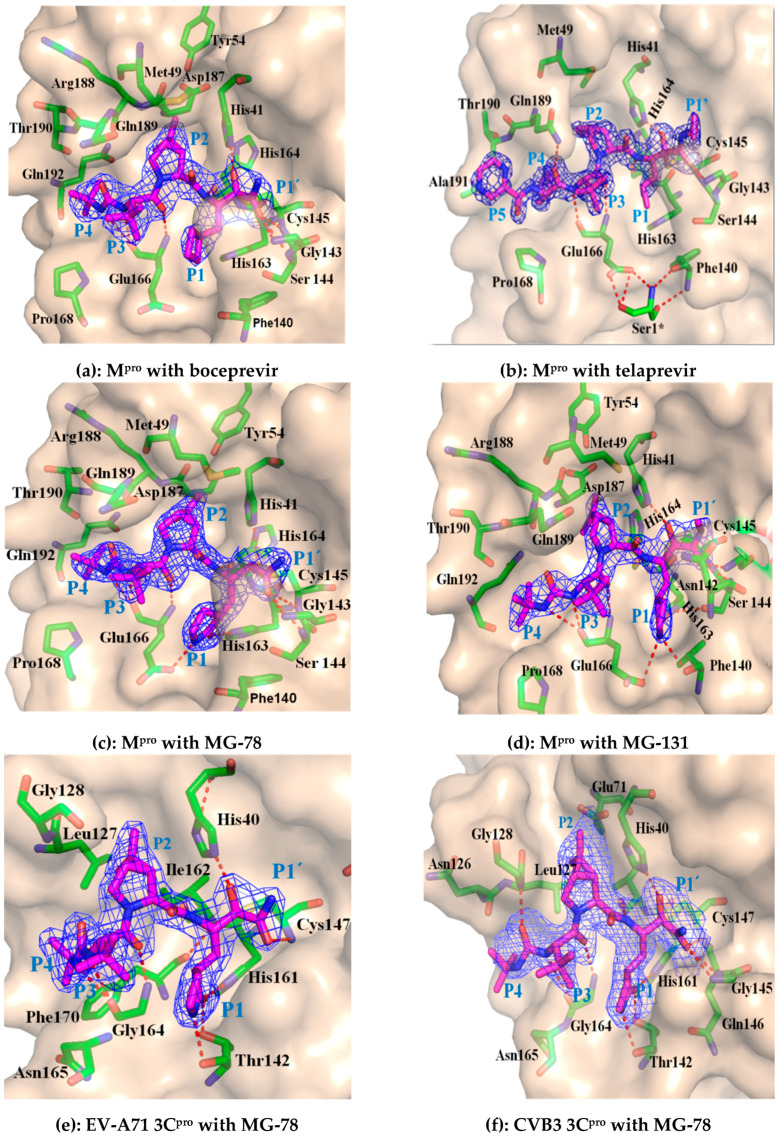
Crystal structures of M^pro^ in complex with boceprevir (**a**), telaprevir (**b**), MG-78 (**c**), and MG-131 (**d**). Crystal structure of EV-A71 3C^pro^ (**e**) and CVB3 3C^pro^ (**f**) in complex with MG-78. The 2Fo-Fc OMIT maps are contoured at 1.5 σ, except for panels (**a** and **f**, which are contoured at 1.0 σ.

As in the boceprevir and telaprevir complexes, the α-carbon of the ketoamide warhead is covalently bound to the Cys145 Sγ of the protease. The oxygen of the thiohemiketal, which is probably anionic at the pH of crystallization of 9.0, accepts a strong hydrogen bond from His41 Nε2 of the catalytic dyad of the protease. The chiral α-C atom is in the S-configuration, just as in the SARS-CoV-2 M^pro^ complex with our compound **13b** [10]. The same arrangement is also found in the complexes between the original target of boceprevir and telaprevir, the HCV NS3/4A protease, and the drugs [16,17] (PDB codes 2CO8, 3SV6). However, it should be noted that the inverse R configuration can also be found, for example in the complex between the α-ketoamide **11a** and the M^pro^s of the human coronavirus NL63 (HCoV NL63) and of SARS-CoV [9] (PDB 6FV2 and 5N19, resp.). The P1′ amide oxygen is fixed in the oxyanion hole by hydrogen bonds from the main-chain amides of Gly143 and Cys145. The extra methyl group of **MG-131** makes a weak (3.3 Å) contact with Nδ2 of Asn142 (Figure 2).

### 2.8. P1 Residue

The main-chain nitrogen of the inhibitors’ P1 building block donates a hydrogen bond to the carbonyl oxygen of His164, an interaction found with nearly all peptidomimetic M^pro^ inhibitors. **MG-78** and **MG-131** make ideal hydrogen bonds to the anchoring points in the S1 pocket, His163, Glu166, and Phe140. In more detail, there is a strong hydrogen bond between the lactam oxygen and His163 Nε2, and a three-center (bifurcated) H-bond between the lactam nitrogen and the Glu166 side-chain carboxylate on the one hand and the main-chain oxygen of Phe140 on the other. The side-chain of Glu166 forms one side-wall of the M^pro^ S1 pocket. Its carboxylic group makes an ion-pair with the N-terminal Ser1* of the other monomer in the dimer, an important interaction contributing to the stability of the M^pro^ dimer [23].

### 2.9. P2 Residue

In the crystal structures of the SARS-CoV-2 M^pro^ complexes with **MG-78** and **MG-131**, the P2 and P3 moieties are virtually superimposable with the corresponding residues in the boceprevir complex (see above). As in most M^pro^ complexes with peptidomimetic compounds, the P2 main-chain oxygen makes no hydrogen bond with the protein but interacts with a water molecule (only weakly seen in the **MG-131** complex, probably because of the lower resolution of the latter crystal structure). Being part of the bicyclic proline derivative, the P2 nitrogen is devoid of H-bonding capabilities. This leaves M^pro^ residue Gln189 without an interaction partner, contributing to its flexibility. In the S2 pocket, the bicyclic proline derivatives make hydrophobic contacts with Met165 (at the bottom of the pocket), Met49 (at the top of the pocket), His41 of the catalytic dyad (side-wall of the pocket), and Gln189 (opposite side-wall; see [1] for a definition of S2 pocket walls).

### 2.10. P3 Position

The P3 main-chain oxygen of **MG-78** and **MG-131** accepts a hydrogen bond from the amide of Glu166 and the P3 amide (part of a urea moiety, see below) interacts with the main-chain carbonyl oxygen of the same residue. These are interactions similarly found in most peptidomimetic inhibitors of coronavirus M^pro^s and hepatitis C virus NS3/4A protease (where boceprevir interacts with main-chain NH and CO of Ala157 [16]). The coronavirus M^pro^s as well as the enterovirus 3C^pro^s and HCV NS3/4A do not have a well-defined S3 pocket and can tolerate many inhibitor moieties here. It has previously been determined through work with a combinatorial library that the best non-proteinogenic amino acid in the P3 position of a tetrapeptidic SARS-CoV-2 M^pro^ substrate is tert-leucine [24]. Boceprevir, telaprevir, **MG-78**, and **MG-131** have exactly this residue in P3; it makes hydrophobic contacts to Met165, the Cβ of Glu166, and the Cγ of Gln189. Because of its free accessibility to bulk solvent due to the lack of an S3 pocket, the P3-tBu group of boceprevir, telaprevir, **MG-78**, and **MG-131** also makes crystal contacts with the α-helical stretch of amino-acid residues Gly#251, Pro#252, and Ala#255 in domain III of a neighboring M^pro^ dimer in the crystal lattice.

### 2.11. P4 Residue

Apart from the P1′ substituent, the only other difference between the **MG-78**/**MG-131** and boceprevir complexes with SARS-CoV-2 M^pro^ concerns the terminal P4-tBu group. In boceprevir, this capping group makes hydrophobic contacts with Leu167 and Pro168, whereas in the **MG-78**/**MG-131** complexes, this same tBu group is in a slightly different orientation and interacts mainly with Thr190. In agreement with this flexibility, the electron density of such terminal P4 groups is often weak in M^pro^ inhibitor complexes, indicating less-than-full occupancy of the major conformation. In boceprevir, **MG-78**, and **MG-131**, the tBu capping group is linked to P3 through a urea moiety, both the P4 and the P3 nitrogen of which donate hydrogen bonds to the Glu166 main-chain carbonyl. The urea oxygen is not involved in specific interactions in any of these M^pro^ structures.

### 2.12. Inhibitory Activity of ***MG-78*** against the M^pro^ of HCoV NL63

Inhibitory activity of SARS-CoV-2 M^pro^ inhibitors against the orthologous protease of alphacoronaviruses would be desirable. We therefore determined the IC_50_ of **MG-78** versus the HCoV-NL63 M^pro^, using the same FRET substrate as for the SARS-CoV-2 enzyme. We had previously shown that this substrate is efficiently cleaved by the HCoV-NL63 M^pro^ [9]. The IC_50_ of **MG-78** was found to be 0.33 ± 0.07 μM, i.e., by a factor of 25 larger than that determined for SARS-CoV-2 M^pro^. Yet, based on these results, **MG-78** is probably a pan-coronavirus inhibitor, although more tests, e.g., against the M^pro^s from MERS-CoV, SARS-CoV, and HCoV 229E, need to be performed. The reduced efficiency of **MG-78** against the HCoV-NL63 M^pro^ is likely caused by the replacement of Gln189 of betacoronavirus M^pro^s by Pro in alphacoronavirus M^pro^s. As we pointed out previously [9], the concomitant loss of plasticity of the S2 pocket of the enzyme reduces its adaptability to the size of the P2 substituent.

### 2.13. Is ***MG-78*** Also an Inhibitor of Enterovirus 3C Proteases?

We had previously shown that enterovirus 3C proteases have a substrate-binding site featuring some similarities with coronavirus main proteases, although their S2 subsite tends to be more open [1]. Leucine is the ideal P2-amino-acid residue in M^pro^ substrates [24], whereas most 3C^pro^s favor Phe in this position. The bicyclic proline derivative of **MG-78** is slightly more voluminous compared to leucine and therefore we wanted to find out whether the compound can also inhibit the 3C^pro^ of enterovirus A71 (EV-A71) and Coxsackievirus B3 (CVB3). Using 10 µM Dabcyl-KEALFQ↓GPPQFE(-Edans)-NH_2_ (↓ indicates the cleavage site) as a FRET substrate, we found IC_50_ values for **MG-78** of 6.7 ± 2.2 μM against the EV-A71 3C^pro^ and 1.45 ± 0.40 μM against CVB3 3C^pro^. To identify the structural basis of these relatively moderate inhibitory activities, we determined crystal structures for both **MG-78** complexes with these enterovirus proteases.

### 2.14. Binding of ***MG-78*** to the Enterovirus A71 and the Coxsackievirus B3 3C Proteases

Crystals of the EV-A71 3C^pro^ complex with **MG-78** were grown in cubic space group P432 and yielded useful diffraction data extending to a Bragg spacing of 2.07 Å, whereas the CVB3 3C^pro^ complex gave crystals in space group C2 that diffracted to 1.65 Å. Compound **MG-78** binds along the substrate-binding cleft in both proteases. Similar to the situation in the coronavirus M^pro^, the nucleophilic attack of the catalytic residue (here Cys147) onto the α-keto group of the α-ketoamide warhead leads to the formation of a thiohemiketal, with a covalent bond between the sulfur of the catalytic Cys and the α-carbon. The oxyanion of the thiohemiketal accepts a hydrogen bond from the general base of the catalytic reaction, His40. The carbon of the thiohemiketal moiety is in the S confi-guration. The amide oxygen of the warhead accepts hydrogen bonds from the Gly145, Gln146, and Cys147 amides of the oxyanion hole. 

### 2.15. P1 Residue

The main-chain amide of the P1 residue donates a hydrogen bond to the carbonyl oxygen of Ile (CVB3: Val) 162. The carbonyl of the γ-lactam accepts hydrogen bonds from His161 Nε2 and the side-chain hydroxyl of Thr142. The lactam nitrogen donates a hydrogen bond to the carbonyl of Thr142.

### 2.16. P2 Residue

The P2 carbonyl oxygen fails to make any interactions with the protein. With regard to the interaction between the bicyclic proline moiety of **MG-78** and the proteases, there are some important differences between the EV-A71 and the CVB3 enzyme. In the EV-A71 3C^pro^ structure, the gem-dimethyl groups of the cyclopropyl proline make contacts with the side-chains of Leu127 and Ser128 of the “beta-ribbon” [25] of the protease. In particular the latter interaction, with a distance of 3.3 Å between one of the methyl groups and the Oγ of Ser128, has to be considered energetically unfavorable and may well constitute one of the reasons for the limited inhibitory potency of compound **MG-78**. The same could be true for the short distance (3.3 Å) between the Cγ atom of the proline and the same Ser128 side-chain. In the CVB3 3C^pro^ structure, the contacts to Leu127 and Gly128 are more relaxed. Instead, there is a rather short distance to the Arg39 guanidinium group.

### 2.17. P3 and P4 Residues

The main-chain oxygen of the P3 residue accepts a hydrogen bond from the amide of Gly164. In the EV-A71 3C^pro^ structure, but not in the CVB3 structure, the P3 tert-butyl side-chain makes contacts to the ligand in a neighboring protease molecule in the crystal lattice. The two nitrogens of the P4/P3 urea unit donate hydrogen bonds to the oxygen of Gly164 in the EV-A71 structure, whereas in the CVB3 protease, only one of these is strong enough to be called a hydrogen bond. The urea oxygen is involved in a water-mediated interaction with the main-chain nitrogen of Ser (CVB3: Gly) 128. This is different from the complexes of **MG-78**, **MG-131**, and boceprevir with the SARS-CoV-2 M^pro^, where the urea oxygen is devoid of any hydrogen-bonding interactions. The terminal tert-butyl group makes weak interactions with Leu125, Leu127, and Asn165 in the EV-A71 structure and with Asn126 in the CVB3 complex.

## 3. Discussion

At the beginning of the current COVID-19 pandemic, we reported the crystal structure of the SARS-CoV-2 main protease (M^pro^) and its complex with a potent α-ketoamide inhibitor, **13b**, that we had designed previously but improved on the basis of this structure [10]. In our original publication, the IC_50_ of compound **13b** was given as 0.67 μM, but after we optimized our assay conditions, this value is now 0.38 μM for the mixture of diastereomers (see Materials and Methods for the optimized conditions).

The potency of irreversible inhibitors is time-dependent in principle, in particular for slowly binding compounds. Apart from the warhead, the decrease of binding entropic penalty by the rigidity of the inhibitors and non-covalent interactions, such as Van der Waals, electrostatic, and hydrophobic interactions and hydrogen bonds, also contribute to the binding [16,26]. Thus, when a slowly binding warhead is coupled to structural elements that confer high affinity, as in the case of the optimized ketoamides studied here, inhibitor-enzyme complexes are formed rapidly [27]. Because we did not observe changes in reaction rates between 1–15 min after substrate addition to a preformed enzyme-inhibitor complex, we regard IC_50_ measurements as sufficient to determine the inhibitory activity of the compounds. This is in line with recent studies [1,12,28] for similar covalently binding α-ketoamide inhibitors. 

Because of the α-ketoamide warhead of **13b**, we were interested in two other α-ketoamide drugs, boceprevir and telaprevir. These are inhibitors of the hepatitis C virus (HCV) NS3/4A protease and have been approved for the treatment of Hepatitis C. In spite of the fact that NS3/4A is a serine protease, whereas the M^pro^ has an active cysteine residue, and in spite of clear differences in amino-acid sequence and three-dimensional structure, we tested whether these compounds are inhibitors of the SARS-CoV-2 M^pro^. We found that boceprevir has moderate activity (IC_50_ = 4.1 μM), whereas telaprevir cannot be called an inhibitor of the enzyme (IC_50_ = 19 μM). We determined crystal structures for either compound in complex with the SARS-CoV-2 M^pro^ and found that boceprevir fits relatively well to the substrate-binding site of the M^pro^, whereas telaprevir had to undergo some conformational changes of the bicyclic proline derivative (proline with an annealed cyclopentane ring) in the P2 position to fit the S2 pocket of the M^pro^. It was probably these enthalpy-costly conformational adjustments as well as an unfavorable contact between the P1′ cyclopropyl ring and the main-chain oxygen of residue Thr26 that led to the inferior inhibitory potency of telaprevir against the SARS-CoV-2 M^pro^, compared to boceprevir. Other groups have also reported crystal structures of the SARS-CoV-2 M^pro^ with boceprevir [11,12,13,14] or telaprevir [11,13,14], but in most of these studies, the conformations of the inhibitors in the coronavirus M^pro^ complexes were not compared with those in the HCV NS3/4A protease complexes.

In the P1 position, boceprevir carries a cyclobutylmethyl moiety and telaprevir an *n*-propyl residue. These have been optimized for interaction with the hydrophobic S1 pocket of the HCV NS3/4A protease, but are unable to provide much binding enthalpy in the complexes with the SARS-CoV-2 M^pro^. Therefore, we replaced this boceprevir residue by the γ-lactam-containing P1 residue that serves as a glutamine surrogate in inhibitors of coronavirus M^pro^ [10] and enterovirus 3C protease [29,30]. The resulting compound, **MG-78**, showed strong potency as an M^pro^ inhibitor, with IC_50_ = 13 nM. The same held true when we added a methyl group in the P1′ position (compound **MG-131**).

Qian et al. [13] as well as Xia et al. [28] followed an approach similar to ours and replaced the P1 residues of both boceprevir and telaprevir with the canonical γ-lactam moiety. However, they used an aldehyde as the warhead of their inhibitors. Qian et al. found that both boceprevir- and telaprevir-derived aldehydes were roughly equipotent inhibitors of the SARS-CoV-2 M^pro^. This is perhaps not surprising, as we have previously shown that the strongly electrophilic aldehyde can overrule the specificity requirements of the coronavirus main protease [31].

Owen et al. [32] also started from the boceprevir scaffold, but they replaced the α-ketoamide warhead by a nitrile. Their compound, PF-07321332 (nirmatrelvir), features the P1 γ-lactam and, identical to boceprevir, the P2 *gem*-dimethyl cyclopropyl proline as well as the P3 *tert*-leucine. This P3 moiety was previously shown by Rut et al. [24] to be ideal in peptidomimetic M^pro^ inhibitors. In the P4 position, nirmatrelvir has a trifluoromethyl residue, which probably contributes to the oral availability of the compound. Nirmatrelvir has been approved by both FDA and EMA and has been introduced into the market as a combination with ritonavir (as a booster) under the name paxlovid.

We had previously synthesized compound **XDL2a** (Figure 1), which is an α-ketoamide carrying the canonical P1-γ-lactam and the P2-bicyclic proline derivative but a cinnamoyl group in P3 (Lin et al., unpublished). This compound was designed as an extension of our cinnamoyl-containing α-ketoamides [9]. Interestingly, this compound was completely inactive in our SARS-CoV M^pro^ assay, preventing us from exploring boceprevir-derivatives as M^pro^ inhibitors at a much earlier point in time.

The coronavirus M^pro^ displays limited relationships to the 3C protease (3C^pro^) of enteroviruses such as enterovirus A71 (EV-A71), the cause of hand, foot and mouth disease (HFMD), and Coxsackievirus B3 (CVB3), which can lead to acute or chronic myocarditis. Members of the two families of proteases employ a cysteine nucleophile and share a specificity for cleaving the peptide P2-P1-P1′-P2′ sequence (hydrophobic a.a.—Gln ↓ small a.a.—small a.a. (M^pro^)/Pro (3C^pro^); ↓ marks the cleavage site), with the hydrophobic P2 residue being of medium size (preferably, Leu) for coronavirus M^pro^s and large size (preferably, Phe) for enterovirus 3C^pro^s. On the other hand, the coronavirus M^pro^s depend on homodimerization for catalytic activity [33], whereas enterovirus 3C^pro^s work as monomers. Also, whereas both families have two β-barrel domains harboring the catalytic site between them, the coronavirus M^pro^ monomer has an additional α-helical domain that is involved in regulation of dimerization [33,34]. Finally, coronavirus M^pro^s feature a catalytic dyad comprising a Cys…His pair, whereas enterovirus 3C^pro^s have a catalytic triad consisting of Cys…His…Glu(Asp). In view of these differences, the alternative designation of the coronavirus M^pro^s as “3C-like proteases” (3CL^pro^s) may be misleading. Even worse, the term “3CL^pro^” is often translated in the literature as “3-chymotrypsin-like”, which makes no sense at all (there is no protease called “3-chymotrypsin”).

In spite of all these differences, we thought that the related architecture of the substrate-binding sites of M^pro^s and 3C^pro^s might allow design and development of inhibitors targeting both families of enzymes with approximately equal potency, as a prerequisite to achieving real broad-spectrum antivirals capable of controlling coronavirus and enterovirus infections. Our motivation for this approach was that prior to the year 2020, there was little interest in antivirals controlling coronaviruses, and the burden of disease caused by enteroviruses was much more substantial (hand-, foot- and mouth-disease caused by enterovirus A71 or Coxsackievirus A16, myocarditis caused by Coxsackievirus B3, pancreatitis caused by Coxsackievirus B4, respiratory diseases caused by enterovirus D68 or human rhinoviruses; see [35] for a review). We designed and synthesized peptidomimetics of the general structure cinnamoyl—P2—γ-lactam—α-ketoamide—P1′, some of which (in particular compound **11r**) turned out to have broad-spectrum activity versus betacoronaviruses and enteroviruses [9]. With the arrival of the COVID-19 pandemic, there was no further need to seek compromises between the steric requirements of the coronavirus M^pro^ and the enterovirus 3C^pro^. Yet, we considered it worthwhile to assess the activity of our novel M^pro^ inhibitor **MG-78** versus enterovirus 3C proteases. However, with IC_50_ values against two representative enterovirus 3C^pro^s larger by more than two orders of magnitude compared to the one versus the SARS-CoV-2 M^pro^, the compound cannot be considered a broad-spectrum inhibitor, at least not of EV-A71 3C^pro^. We found the same for Pfizer’s nirmatrelvir (the anticoronaviral component of paxlovid), for which we determined inhibition rates of 33% and 55% against the 3C protease of EV-A71 and CVB3, respectively (not shown). Whereas the crystal structure of **MG-78** in complex with the SARS-CoV-2 M^pro^ revealed an ideal fit, those of the complexes with CVB3 3C^pro^ and EV-A71 3C^pro^ demonstrated some steric incompatibilities that likely reduce the inhibitory potency. Such incompatibilities are more serious in the case of the EV-A71 3C^pro^ compared to the CVB3 3C^pro^ complex, in remarkable correlation with the inhibitory activities of **MG-78** against the two proteases.

## 4. Conclusions

We used our crystal structures of the SARS-CoV-2 M^pro^ in complexes with the approved HCV drugs, boceprevir and telaprevir, to design two improved α-ketoamides, **MG-78** and **MG-131**, starting from boceprevir. Both of these compounds carry a γ-lactam in the P1 moiety, a group typical of M^pro^ inhibitors, instead of the cyclobutyl of boceprevir. The compounds display very strong inhibitory activity versus the SARS-CoV-2 M^pro^ and good activity against the M^pro^ of the alphacoronavirus HCoV NL63, so they can be assumed to be pan-coronavirus inhibitors. However, they show only moderate inhibitory activity against the 3C proteases of the enteroviruses CVB3 and EV-A71.

## 5. Materials and Methods

### 5.1. Inhibitors

Boceprevir was purchased from Selleckchem. Telaprevir for biochemical experiments was from Selleckchem; for X-ray crystallography, material from Adooq Bioscience was used. The syntheses of **MG-78** and **MG-131** are described in the Section 2 “Results” and further below. PF-07321332 (nirmatrelvir) was custom-synthesized by Tocris (Bio-Techne), following the protocol in [32].

### 5.2. Recombinant Protein Production

The SARS-CoV-2 M^pro^ was produced as described in [10]. For preparation of the Coxsackievirus B3 3C protease, the CVB3-3Cpro-pET23a plasmid was transformed into BL21 (DE3) competent cells. The 3C^pro^-containing supernatant was injected onto a metal-affinity HisTrap FF column (GE Healthcare) and eluted using a linear gradient with 0 to 100% elution buffer (20 mM Tris-HCl pH 7.8, 500 mM NaCl, 500 mM imidazole) followed by gel filtration chromatography using a HiLoad 16/600 Superdex 75 prep-grade column. The purified CVB3 3C^pro^ was stored at −80 °C in a buffer comprising 20 mM Tris-HCl pH 7.8, 500 mM NaCl.

The EV71-3Cpro-pET16b plasmid was transformed into BL21-Gold (DE3) competent cells. The His-tagged protein was purified by Ni^2^^+^-affinity chromatography with a linear gradient of 0 to 100% elution buffer (25 mM Tris-HCl pH 7.4, 200 mM NaCl, 500 mM imidazole, 5 mM DTT), followed by gel filtration chromatography using a HiLoad 16/600 Superdex 75 prep-grade column pre-equilibrated with 25 mM Tris-HCl, pH 7.4, 200 mM NaCl, and 5 mM DTT.

### 5.3. Determination of the Inhibitory Activity of Compounds against Coronavirus M^pro^s and Enterovirus 3C^pro^s

The inhibitory activities versus the SARS-CoV-2 M^pro^ of boceprevir, telaprevir, **MG-78**, and **MG-131** were determined in a reaction buffer containing 20 mM HEPES, 120 mM NaCl, 0.4 mM EDTA, 20% glycerol, 4 mM DTT (freshly added before the measurements), pH 7.0, at 37 °C. A fluorescent substrate with the cleavage site (indicated by the arrow, ↓) of SARS-CoV-2 M^pro^ (Dabcyl-KTSAVLQ↓SGFRKM-E(Edans)-NH_2_; Biosyntan, Berlin) was used, and the fluorescence signal of the cleaved substrate was monitored using a Tecan Spark^®^ fluorescence spectrophotometer at an emission/excitation wavelength of 460/360 nm. Initially, 10 μL (per well) of the SARS-CoV-2 M^pro^ was pipetted into a 96-well plate with the corresponding drops containing 59 μL reaction buffer (final enzyme concentration: 50 nM). Subsequently, the inhibitors were added to give different final concentrations (0, 0.1, 0.4, 1.2, 3.7, 11, 33, and 100 µM). The inhibitor-M^pro^ mixture was incubated at 37 °C for 10 min. Finally, the reaction was initiated by adding 30 μL of the substrate dissolved in the reaction buffer with the corresponding concentrations described above. The same assay was used for the determination of inhibitory activity of **MG-78** against HCoV-NL63 M^pro^, as this enzyme cleaves the SARS-CoV-2 M^pro^ substrate with high efficiency [9].

To assess the inhibitory activity of **MG-78** versus Coxsackievirus B3 3C^pro^ and entero-virus A71 3C^pro^, the FRET-substrate Dabcyl-KEALFQ↓GPPQF-E(Edans)-NH_2_; Biosyntan, Berlin) was used, and the fluorescence signal was also monitored using a Tecan Spark^®^ fluorescence spectrophotometer with a wavelength of 460/360 nm (emission/excitation). Assay samples contained 59 µL of reaction buffer (20 mM HEPES, 120 mM NaCl, 0.4 mM EDTA, 20% glycerol, 4 mM DTT (freshly added before the measurements), pH 7.0, 10 µL enzyme solution (final concentration of Coxsackievirus B3 3C^pro^ or enterovirus A71 3C^pro^: 1.0 µM).

All reactions were monitored at 37 °C for 15 min. Measurements of enzymatic activity were performed in triplicate and the value was presented as mean ± standard deviation (SD). The initial velocity of the enzymatic reaction with compounds was calculated by linear regression for the first minute of the progression curve. Inhibition in % was obtained as (1 − (V_max_/V_0_)) × 100%. V_0_ is the velocity obtained for the DMSO control (inhibitor concentration of 0 µM).

The log [% inhibition] was plotted against different concentrations of inhibitors (dose-response-inhibition). The IC_50_ values were calculated by non-linear regression (Curve fit: Y: Bottom + (Top-Bottom)/(1 + 10^ ((X-lg IC50))) using GraphPad Prism 9.0.

### 5.4. Crystallization and Diffraction Data Collection of SARS-CoV-2 M^pro^ in Complexes with Boceprevir, Telaprevir, ***MG-78***, or ***MG-131***

The purified SARS-CoV-2 M^pro^ was mixed with boceprevir or telaprevir at the molar ratio of 1:4, and the mixture was incubated in the cold room overnight. The next day, the protein−compound mixture was centrifuged at 13,000 rpm to remove precipitate. The supernatant was subjected to cocrystallization screening with commercially available kits (PACT premier^TM^ HT-96 and Shot Gun (SG1^TM^ Screen, Molecular Dimensions)), using a Gryphon LCP robot (Art Robbins Instruments) and the vapor-diffusion sitting-drop method at 20 °C, with equilibration of 0.15 μL protein−compound solution mixed with 0.15 μL mother liquor against 40 μL reservoir. Crystals were observed under several different conditions for both of the cocrystallization trials with boceprevir and telaprevir. For co-crystallization of the SARS-CoV-2 M^pro^ with **MG-78** or **MG-131**, the protein (at a concentration of 10.5 mg/mL) was incubated overnight with a 5-fold molar excess of inhibitor at 4 °C. Crystals grew at room temperature under condition D6 of the PACT premier^®^ kit from Molecular Dimensions, containing 0.1 M MMT (malic acid—MES—Tris in the ratio 1:2:2), pH 9.0, plus 25% w/v polyethylene glycol 1500.

Crystals were fished from different drops with the corresponding cryo-protectant. Liquid nitrogen was employed for flash-cooling of the fished crystals prior to diffraction data collection. Diffraction data from the complex of M^pro^ with boceprevir were collected from a crystal grown under the condition of Shotgun No. C11 (0.2 M sodium acetate trihydrate, 0.1 M sodium cacodylate pH 6.5, 30% PEG 8000), with the cryo-protectant consisting of mother liquor plus 5% glycerol, and 2 mM boceprevir. Diffraction data for the complex of M^pro^ with telaprevir were collected from a crystal grown under the condition of Shotgun No. C4 (0.2 M potassium sodium tartrate tetrahydrate, 20% PEG 3350), and crystals were cryo-protected with mother liquor plus 20% glycerol, and 2 mM telaprevir. Diffraction data for the complex of M^pro^ with **MG-78** or **MG-131** were collected from crystals grown under the condition mentioned above (D6 of PACT premier^®^). All diffraction data sets were collected at 100 K at DESY PETRA III beamline P11 using a Pilatus 6M detector (Dectris), and with synchrotron radiation of wavelength of 1.0332 Å. 

### 5.5. Crystallization and Diffraction Data Collection of the ***MG-78*** Complexes with Enterovirus-A71 3C^pro^ and Coxsackievirus B3 3C^pro^

The EV-A71 3C^pro^ was concentrated to 7 mg/mL and co-incubated with a 3-fold molar excess of **MG-78** for 2 h at room temperature. Crystals of the enzyme–inhibitor complex grew at 12 °C and were obtained from a solution containing 100 mM Tris-HCl pH 7.0, 34% PEG 3350, and 200 mM sodium citrate.

The CVB3 3C^pro^ was concentrated to 17.5 mg/mL and co-incubated with a 5-fold molar excess of **MG-78** for 2.5 h at room temperature. Crystals of the enzyme–inhibitor complex were obtained from a solution containing 100 mM Tris-HCl pH 8.6, 20% PEG 3350, and 200 mM MgCl_2_.

All diffraction data sets were collected at 100 K at DESY PETRA III beamline P11 using a Pilatus 6M detector (Dectris), and with synchrotron radiation of wavelength of 1.0332 Å.

### 5.6. Diffraction Data Processing, Structure Solution, and Refinement

All data sets were processed using the programs XDSapp [36], Pointless [37,38,39], and scaled by Scala [37,39]. The phase problem was solved by using the molecular replacement method, employing the Molrep program [39,40], and the structure of SARS-CoV-2 M^pro^ free enzyme [10] (PDB code: 6Y2E) was selected as search model. Geometric restraints for the ligands were generated by using the JLigand program [39,41], and the compounds were built into F_o_-F_c_ difference density using the Coot software [42]. The Refmac5 program [43] was used for refinement of the structures.

### 5.7. Compound Synthesis

#### 5.7.1. General Experimental Information

Commercial reagents were used as received, and all other reagents were prepared using known literature procedures. All solvents used for reactions, workups, and purifications had the HPLC purity grade. Dried solvents were purchased in water-free form (99.5%, extra dry, absolute, AcroSeal^TM^, ACROS Organics^TM^) and used as received.

Reactions were either monitored by Liquid Chromatography-coupled Mass Spectrometry (LCMS) analysis or thin-layer chromatography (TLC) on “TLC Silica gel 60 F254” plates (Merck) and visualized by staining with aqueous basic KMnO_4_. LCMS was conducted with an Agilent^®^ 1260 HPLC System with a DAD detector and an Agilent^®^ 6130 quadrupole mass detector with Electrospray Ionization (ESI) (MeCN/H_2_O + 0.1% HCOOH).

Flash chromatography was performed on silica gel 60 (technical grade, pore size 60 Å, 40–63 µm, 230–400 mesh, Supelco^®^). Reverse-Phase High Pressure Liquid Chromatography (RP-HPLC) was performed with a Phenomenex Luna C18 RP-column 00G-4252-P0-AX, 5 µm, 100 Å, 250×21.2 mm (flow rate 10 mL/min, max. loading 100 mg crude) coupled to a Thermo Fisher Scientific^®^ Dionex Ultimate 3000 HPLC-System using a ternary solvent mixture (MeCN/H_2_O + 0.1% HCOOH). Product-containing fractions were identified by LCMS and lyophilized to dryness.

For Nuclear Magnetic Resonance (NMR) spectroscopic analysis, Bruker Avance III or Bruker Avance III HD spectrometers were employed and ^1^H NMR spectra were recorded at 400 MHz, 500 MHz, or 700 MHz, respectively 126 MHz and 176 MHz for ^13^C NMR spectra. Chemical shifts are reported in parts per million (ppm) using the residual non-deuterated solvent resonance for proton measurements and the solvent resonance for carbon measurements as internal standards (D_3_COD: ^1^H = 3.31 ppm and ^13^C = 49.00 ppm; DMSO-d_6_: ^1^H = 2.50 ppm and ^13^C = 39.52 ppm). Data are reported as follows: chemical shift (multiplicity (s = singlet, d = doublet, t = triplet, q = quartet, m = multiplet), coupling constant(s) in Hz, integration).

High resolution mass spectrometry (HRMS) was performed using a Dionex Ultimate 3000 HPLC system equipped with a DAD detector and a Bruker maXis HD QTOF mass detector with electrospray ionization (ESI). Samples were injected via an Ultimate 3000RS autosampler (Thermo Fisher Scientific) and data are reported in the form of mass-to- charge ratio (*m*/*z*).

#### 5.7.2. Preparation of tert-Butyl ((2S)-1-cyano-1-hydroxy-3-((S)-2-oxopyrrolidin-3-yl)propan-2-yl)carbamate (**2_a,b_**)

*tert*-Butyl ((*S*)-1-oxo-3-((*S*)-2-oxopyrrolidin-3-yl)propan-2-yl)carbamate (**1**) (297 mg, 1.159 mmol, 1.00 eq) was dissolved in anhydrous CH_2_Cl_2_ (3 mL) under an inert gas atmosphere. Et_3_N (201 μL, 1.448 mmol, 1.25 eq) was added, followed by the addition of acetone cyanohydrin (159 μL, 1.738 mmol, 1.50 eq). The reaction mixture was stirred at ambient temperature for 14 h and concentrated at 40 °C to a final pressure of 2 mbar. The crude reaction mixture was redissolved in CH_2_Cl_2_ (5 mL) and the volatiles were removed under reduced pressure to yield a slightly yellow colored foam. Purification by flash chromatography (12.5 -> 15% *i*-PrOH/CH_2_Cl_2_) furnished the title compound **2_a,b_** (277 mg, 84%) as a white foam.

ESI-HRMS: calc. for C_13_H_21_N_3_NaO_4_ [M + Na]^+^: *m*/*z* = 306.1424, found *m*/*z* = 306.1421.


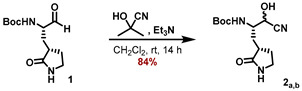


#### 5.7.3. Preparation of tert-Butyl ((2S)-1-cyano-1-hydroxy-3-((S)-2-oxopyrrolidin-3-yl)propan-2-yl)carbamate (**3_a,b_**)

To a solution of *tert*-butyl ((2S)-1-cyano-1-hydroxy-3-((S)-2-oxopyrrolidin-3-yl) propan-2-yl)carbamate (**2_a,b_**) (275 mg, 96.06 μmol, 1.00 eq) in MeOH (4 mL) was added H_2_O_2_ (0.8 mL), followed by the addition of LiOH (27.43 mg, 114.53 μmol, 1.18 eq). The reaction mixture was stirred for 4 h at 0 °C, saturated aqueous Na_2_S_2_O_3_ solution was added (50 mL), and the mixture was extracted with EtOAc (4 × 75 mL). After drying of the combined organic extracts with Na_2_SO_4_ and removal of all volatiles, the crude product was purified by HPLC to provide both diastereomers of the *α*-hydroxyamide **3** (36.0 mg of major diastereomer **3_a_**, 30.1 mg of minor diastereomer **3_b_**, 23% for sum of both) as white solids.


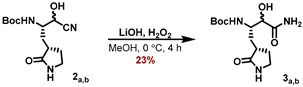


#### 5.7.4. Major Diastereomer **3_a_**

**^1^H NMR (500 MHz, D_3_COD, 298 K):** *δ* = 4.14 (d, *J* = 2.3 Hz, 1 H), 3.99 (dt, *J* = 12.2 Hz, 2.3 Hz, 2.3 Hz, 1 H), 3.35–3.26 (m, 2 H), 2.37(m_c_, 2 H), 2.05 (m_c_, 1 H), 1.75 (m_c_, 1 H), 1.45 (s, 9 H), 1.23 (m_c_, 1 H) ppm.

**^13^C NMR (125 MHz, D3COD, 298 K):** δ = 182.8, 177.6, 158.0, 80.3, 75.0, 52.6, 41.5, 39.6, 31.1, 28.7, 28.6 ppm.

#### 5.7.5. Minor Diastereomer **3_b_**

**^1^H NMR (500 MHz, D_3_COD, 298 K):** *δ* = 4.02 (m_c_, 1 H), 3.98 (d, *J* = 2.4 Hz, 1 H), 3.38–3.27 (m, 2 H), 2.42 (m_c_, 2 H), 2.12 (m_c_, 1 H), 1.84 (m_c_, 1 H), 1.49–1.39 (m, 10 H).

**^13^C NMR (125 MHz, D_3_COD, 298 K):** *δ* = 182.7, 178.2, 158.0, 80.2, 74.7, 52.5, 41.5, 39.9, 34.9, 28.9, 28.7 ppm.

**ESI-HRMS:** calc. for C_13_H_24_N_3_O_5_ [M + H]+: *m*/*z* = 302.1711, found *m*/*z* =302.1712.

#### 5.7.6. Preparation of (3S)-3-Amino-2-hydroxy-4-((S)-2-oxopyrrolidin-3-yl)butanamide (**4_a_**)

The major diastereomer of the Boc-protected *α*-hydroxyamide **3_a_** (34 mg, 112 μmol, 1.00 eq) was suspended in a 4 m solution of HCl in 1,4-dioxane (6 mL) and the mixture was stirred for 2 h. All volatiles were removed under reduced pressure, 1,4-dioxane (5 mL) was added, and the suspension was again concentrated to dryness to yield the resulting ammonium salt **4a** (26.8 mg, >99%), which was used for the next step without further purification.

**ESI-HRMS:** calc. for C_8_H_16_N_3_O_3_ [M + H]^+^: *m*/*z* = 202.1186, found *m*/*z* = 202.1186.


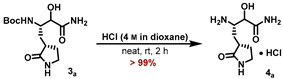


#### 5.7.7. Preparation of (1R,2S,5S)-N-((2S)-4-Amino-3-hydroxy-4-oxo-1-((S)-2-oxopyrrolidin-3-yl) butan-2-yl)-3-((S)-2-(3-(tert-butyl)ureido)-3,3-dimethylbutanoyl)-6,6-dimethyl-3-azabicyclo[3.1.0]hexane-2-carboxamide (**6**)

A 2 dram vial, equipped with a stirring bar, was charged with (1*R*,2*S*,5*S*)-3-((*S*)-2-(3-(*tert*-butyl)ureido)-3,3-dimethylbutanoyl)-6,6-dimethyl-3-azabicyclo[3.1.0]hexane-2-carboxylic acid (*5*) (46.38 mg, 126.2 μmol, 1.25 eq), (2*S*)-4-amino-3-hydroxy-4-oxo-1-((*S*)-2-oxopyrrolidin-3-yl)butan-2-aminium hydrochloride (24.0 mg, 100.98 μmol, 1.00 eq) and HATU (51.83 mg, 136.32 μmol, 1.35 eq) purged with Ar (balloon, 5 min) and chilled in an ice bath. DMF (0.8 mL) was added, followed by the addition of DIPEA (58.0 μL, 333.22 μmol, 3.30 eq) and the mixture was stirred at 0 °C for 3 h. HPLC purification of the crude reaction mixture yielded the title compound 6 (30.2 mg, 54.8 μmol, 56%).

**^1^H NMR (700 MHz, D_3_COD, 298 K):** *δ* = 4.33 (m_c_, 1 H), 4.30 (s, 1 H), 4.26 (s, 1 H), 4.10 (d, *J* = 3.9 Hz, 1 H), 4.03 (d, *J* = 10.3 Hz, 1 H), 3.96 (dd, *J* = 10.3 Hz, 5.5 Hz, 1 H), 3.26 (m_c_, 1 H), 3.22 (m_c_, 1 H), 2.62 dddd, *J* = 11.9 Hz, 10.3 Hz, 8.6 Hz, 3.5 Hz, 1 H), 2.32 (m_c_, 1 H), 2.17 (m_c_, 1 H), 1.68 (dq, *J* = 12.5 Hz, 9.2 Hz, 1 H), 1.56 (dd, *J* = 7.7 Hz, 5.5 Hz, 1 H), 1.44 (d, *J* = 7.7 Hz, 1 H), 1.29 (m_c_, 1 H), 1.25 (s, 9 H), 1.06 (s, 3 H), 0.99 (s, 9 H), 0.94 (s, 3 H) ppm.

**^13^C NMR (176 MHz, D_3_COD, 298 K):** *δ* = 182.8, 177.5, 173.8, 173.4, 159.7, 74.7, 62.1, 58.9, 51.0, 50.7, 49.3, 41.4, 39.2, 35.8, 32.3, 31.0, 29.6, 29.3, 28.9, 27.0, 26.5, 20.4, 13. ppm.

**ESI-HRMS:** calc. for C_27_H_47_N_6_O_6_ [M + H]^+^: *m*/*z* = 551.3552, found *m*/*z* =551.3545.


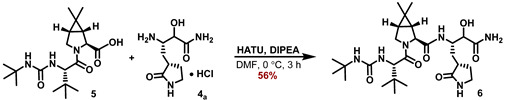


#### 5.7.8. Preparation of (1R,2S,5S)-N-((S)-4-Amino-3,4-dioxo-1-((S)-2-oxopyrrolidin-3-yl)butan2-yl)-3-((S)-2-(3-tert-butylureido)-3,3-dimethylbutanoyl)-6,6-dimethyl-3-azabicylo[3.1.0]hexane-2-carboxamide (MG-78)

The *α*-hydroxy-ketoamide **6** (29.0 mg, 52.7 μmol, 1.00 eq) was dissolved in a 2:1 mixture of MeCN/CH_2_Cl_2_ (2 mL) and NaHCO_3_ (1.77 mg, 21.1 μmol, 0.40 eq) and DMP (27.3 mg, 64.3 μmol, 1.22 eq) was added. After stirring the reaction mixture for 1.5 h, another portion of DMP (11.2 mg, 26.3 μmol, 0.50 eq) was added. After 1.5 h, *i*-PrOH (20 µL) was added, and the reaction mixture was stirred for further 10 min. Then the suspension was filtered through a cotton plug in a Pasteur pipette onto a flash column. Elution with MeOH/CH_2_Cl_2_ (3 -> 10%) and removal of the solvent provided the title compound **MG-78** as a white solid (20.1 mg, 36.6 μmol, 70%).

**^1^H NMR (400 MHz, DMSO-d_6_, 298 K):** *δ* = 8.50 (d, *J* = 8.1 Hz, 1 H), 8.05 (s, 1 H), 7.79 (s, 1 H), 7.59 (s, 1 H), 5.93 (s, 1 H), 5.84 (d, *J* = 9.9 Hz, 1 H), 5.14 (ddd, *J* = 11.6 Hz, 8.3 Hz, 3.1 Hz, 1 H), 4.24 (s, 1 H), 4.10 (d, *J* = 10.4 Hz, 1 H), 4.08 (d, *J* = 10.5 Hz, 1 H) 3.93 (d, *J* = 10.4 Hz, 1 H), 3.78 (dd, *J* = 10.2 Hz, 5.4 Hz, 1 H), 3.15 (t, *J* = 9.1 Hz 1 H), 3.05 (m_C_, 1 H), 2.18 (m_C_, 1 H), 1.85 (m_C_, 1 H), 1.64 (m_C_, 1 H), 1.56 (m_C_, 1 H), 1.46 (dd, *J* = 7.6 Hz, 5.6 Hz, 1 H), 1.24 (d, *J* = 7.6 Hz, 1 H), 1.16 (s, 9 H), 1.01 (s, 3 H), 0.89 (s, 9 H), 0.86 (s, 3 H).

**^13^C NMR (100 MHz, DMSO-d_6_, 298 K):** *δ* = 197.4, 178.2, 171.2, 170.8, 163.0, 157.4, 59.5, 56.8, 51.3, 48.9, 47.4, 37.3, 34.0, 31.7, 30.6, 29.1, 27.29, 27.27, 26.4, 26.0, 18.6, 12.6 ppm.

**ESI-HRMS:** calc. for C_27_H_44_N_6_NaO_6_ [M + Na]^+^: *m*/*z* = 571.3215, found *m*/*z* = 571.3226.


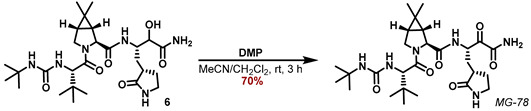


#### 5.7.9. Preparation of (3S)-3-((tert-Butoxycarbonyl)amino)-1-(methylamino)-1-oxo-4-((S)2-oxopyrrolidin-3-yl)butan-2-yl acetate (**7_a,b_**)

*tert*-Butyl ((*S*)-1-oxo-3-((*S*)-2-oxopyrrolidin-3-yl)propan-2-yl)carbamate (**1**) (295 mg, 1.15 mmol, 1.00 eq) was dissolved in anhydrous CH_2_Cl_2_ (5 mL) under an inert gas atmosphere. Methyl isocyanide (80.9 μL, 1.36 mmol, 1.18 eq) was added, followed by the addition of AcOH (77.8 μL, 1.36 mmol, 1.18 eq), and the reaction mixture was stirred at ambient temperature for 14 h. After removal of all volatiles, the crude reaction mixture was purified by flash chromatography (5 -> 10% MeOH/CH_3_Cl) to yield the title compounds **7_a,b_** as an inconsequential mixture of diastereomers in form of a colorless foam (363 mg, 1.016 mmol, 88%).

**ESI-HRMS:** calc. for C_16_H_27_N_3_NaO_6_ [M + Na]^+^: *m*/*z* = 380.1792, found *m*/*z* = 380.1789.


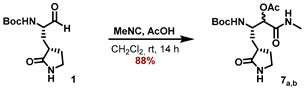


#### 5.7.10. Preparation of tert-Butyl ((2S)-3-hydroxy-4-(methylamino)-4-oxo-1-((S)-2-oxo-pyrrolidin-3-yl)butan-2yl)carbamate (**8_a,b_**)

A solution of the *α*-acetoxy amides **7_a,b_** (350 mg, 979 µmol, 1.00 eq) in MeOH (15 mL) was diluted with water (7.5 mL). After addition of LiOH (40.2 mg, 1.66 mmol, 1.70 eq), the reaction mixture was stirred for 3 h or till TLC showed complete consumption of the starting material. The reaction mixture was adjusted to pH = 7 with 1 M HCl and most of the MeOH was removed under reduced pressure. The remaining mixture was partitioned between brine (30 mL) and EtOAc (50 mL) reaction and the aqueous phase was further extracted with EtOAc (2 × 50 mL). Drying of the combined organic extracts with Na_2_SO_4_ and removal of all volatiles yielded the title compounds **8_a,b_** as an amorphous colorless solid (308 mg, 978 μmol, >99%), which was used in the next step without further purification.


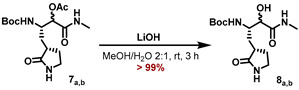


#### 5.7.11. Major Diastereomer **8_a_**

**^1^H NMR (700 MHz, DMSO-d_6_, 298 K):** *δ* = 7.78 (dd, *J* = 9.3 Hz, 4.7 Hz, 1 H), 7.55 (s, 1 H), 6.10 (d, *J* = 9.7 Hz, 1 H), 5.73 (s, 1 H), 3.83 (d, *J* = 3.4 Hz, 1 H), 3.76 (m_c_, 1 H), 3.10 (m_c_, 2 H), 2.57 (d, *J* = 4.7 Hz, 3 H), 2.23 (m_c_, 1 H), 2.10 (m_c_, 1 H), 1.79 (m_c_, 1 H), 1.61 (m_c_, 1 H), 1.35 (s, 9 H), 1.25 (m_c_, 1 H) ppm.

**^13^C NMR (176 MHz, DMSO-d_6_, 298 K):** *δ* = 178.7, 172.6, 155.3, 77.8, 73.0, 51.3, 39.5, 37.9, 32.9, 28.2, 27.7, 25.3 ppm.

**ESI-HRMS:** calc. for C_14_H_27_N_3_O_5_ [M + H]^+^: *m*/*z* = 316.1867, found *m*/*z* = 316.1867.

#### 5.7.12. Preparation of (3S)-3-Amino-2-hydroxy-N-methyl-4-((S)-2-oxopyrrolidin-3-yl)butanamide (**9_a,b_**)

*tert*-Butyl ((2*S*)-3-hydroxy-4-(methylamino)-4-oxo-1-((*S*)-2-oxopyrrolidin-3-yl)butan2yl)carbamate (**8_a,b_**) (170 mg, 539 µmol, 1.00 eq) was treated with 4 m HCl in 1,4-dioxane (10 mL), and the reaction mixture was stirred for 3 h or till all starting material was consumed. The resulting suspension was concentrated to dryness, 1,4-dioxane (10 mL) was added, the solid was resuspended, and all volatiles were removed again. This procedure was repeated twice by using MTBE (10 mL) for resuspension to yield the title compound as a colorless solid (135 mg, 536 μmol, > 99%), which was used in the next step without further purification. 

**ESI-HRMS:** calc. for C_9_H_18_N_3_O_3_ [M + H]^+^: *m*/*z* = 216.1343, found *m*/*z* = 216.1341.


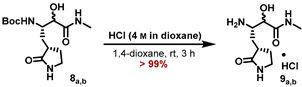


#### 5.7.13. Preparation of (1R,2S,5S)-3-((S)-2-(3-(tert-Butyl)ureido)-3,3-dimethylbutanoyl)-N((2S)3-hydroxy4-(methylamino)-4-oxo-1-((S)-2-oxopyrrolidin-3-yl)butan-2-yl)-6,6-dimethyl -3-azabicyclo[3.1.0]hexane-2-carboxamide (**10_a,b_**)

A 25 mL flask was charged with (1*R*,2*S*,5*S*)-3-((*S*)-2-(3-(*tert*-butyl)ureido)-3,3-dimethylbutanoyl)-6,6-dimethyl-3-azabicyclo[3.1.0]hexane-2-carboxylic acid (124.6 mg, 387 μmol, 1.15 eq), (2*S*)-4-amino-3hydroxy4-oxo-1-((*S*)-2-oxopyrrolidin-3-yl)butan-2-aminium hydrochloride (84.6 mg, 336 μmol, 1.00 eq), and HATU (149.5 mg, 393.2 μmol, 1.17 eq), purged with Ar (balloon, 5 min) and chilled in an ice bath. DMF (3 mL) was added, followed by the addition of DIPEA (186 μL, 1.065 mmol, 3.17 eq), and the mixture was stirred at 0 °C for 3 h. The crude reaction mixture product was purified by HPLC to provide both diastereomers of the *α*-hydroxyamides **10_a,b_** (89.9 mg of major diastereomer **10_a_**, 30.1 mg of minor diastereomer **10_b_**, 249 µmol, 74% for sum of both) as white solids.


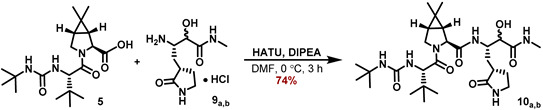


#### 5.7.14. Major Diastereomer **10_a_**

**^1^H NMR (500 MHz, D_3_COD, 298 K):** *δ* = 4.40 (dt, *J* = 12.5 Hz, 2.8 Hz, 1 H), 4.27–4.24 (m, 2 H), 4.02 (s, 1 H), 4.01 (d, *J* = 8.5 Hz, 1 H), 3.94 (dd, *J* = 10.2Hz, 5.5 Hz, 1 H), 3.28 (td, *J* = 9.5 Hz, 1.9 Hz, 1 H), 2.74 (s, 3 H), 2,58 (m_c_, 1 H), 2.41 (m_c_, 1 H), 2.26 (m_c_, 1 H), 1.78 (dq, *J* = 12.5 Hz, 9.3 Hz, 1 H), 1.54 (dd, *J* = 7.6 Hz, 5.4 Hz, 1 H), 1.39 (ddd, *J* = 13.9 Hz, 12.0 Hz, 3.1 Hz, 1 H), 1.27–1.23 (m, 10 H), 1.05 (s, 3 H), 0.98 (s, 9 H), 0.93 (s, 3 H) ppm.

**^13^C NMR (126 MHz, D_3_COD, 298 K):** *δ* = 182.8, 175.5, 173.7, 173.4, 159.7, 74.6, 62.1, 58.8, 50.8, 50.7, 49.2, 41.4, 39.5, 35.8, 34.5, 32.6, 29.6, 29.21, 29.19, 27.1, 26.6, 26.0, 20.3, 13.2 ppm.

**ESI-HRMS:** calc. for C_28_H_49_N_6_O_6_ [M + H]^+^: *m*/*z* = 565.3708, found *m*/*z* = 565.3706.

#### 5.7.15. Preparation of (1R,2S,5S)-3-((S)-2-(3-tert-Butylureido)-3,3-dimethylbutanoyl) -6,6-dimethyl-N-((S)-4-(methylamino)-3,4-dioxo-1-((S)-2-oxopyrrolidin-3-yl)butan-2-yl)-3-azabicyclo[3.1.0]hexane-2-carboxamide (MG-131)

The major diastereomer of the *α*-hydroxy-ketoamide **10_a_** (30.1 mg, 53.3 μmol, 1.00 eq) was dissolved in CH_2_Cl_2_ (1 mL) and NaHCO_3_ (1.79 mg, 21.3 μmol, 0.40 eq) and DMP (33.9 mg, 80.0 μmol, 1.50 eq) was added. After stirring the reaction mixture for 3 h or till all starting material was consumed, *i*-PrOH (20 µL) was added, and the reaction mixture was stirred for further 10 min. Then the suspension was filtered through a cotton plug in a Pasteur pipette onto a flash column. Elution with MeOH/CH_2_Cl_2_ (3 -> 10%) and removal of the solvent provided the title compound **MG-131** as a white solid (25.3 mg, 45.0 μmol, 84%).

**^1^H NMR (500 MHz, DMSO-d_6_, 298 K):** *δ* = 8.66 (dd, *J* = 9.6 Hz, 4.7 Hz, 1 H), 8.54 (d, *J* = 8.0 Hz, 1 H), 7.60 (s, 1 H), 5.94 (s, 1 H), 5.84 (d, *J* = 10.0 Hz, 1 H), 5.13 (ddd, *J* = 11.5 Hz, 8.1 Hz, 3.2 Hz, 1 H), 4.24 (s, 1 H), 4.09 (d, *J* = 10.0 Hz, 1 H), 3.92 (d, *J* = 10.3 Hz, 1 H), 3.77 (dd, *J* = 10.3 Hz, 5.5 Hz, 1 H), 3.16 (t, *J* = 9.1 Hz, 1 H), 3.05 (m_C_, 1 H), 2.65 (d, *J* = 4.9 Hz, 3 H), 2.18 (m_C_, 1 H), 1.84 (m_C_, 1 H), 1.66 (m_C_, 1 H), 1.58 (m_C_, 1 H), 1.45 (dd, *J* = 7.6 Hz, 5.5 Hz, 1 H), 1.23 (d, *J* = 7.7 Hz, 1 H), 1.16 (s, 9 H), 1.01 (s, 3 H), 0.89 (s, 9 H), 0.85 (s, 3 H) ppm.

**^13^C NMR (126 MHz, DMSO-d_6_, 298 K):** *δ* = 196.8, 178.1, 171,2, 170.8, 161.3, 157.4, 59.5, 56.8, 51.7, 48.9, 47.4, 37.4, 34.0, 31.7, 30.6, 29.1, 27.28, 27.25, 26.4, 26.1, 25.5, 18.6, 12.6 ppm.

**ESI-HRMS:** calc. for C_28_H_47_N_6_O_6_ [M + H]^+^: *m*/*z* = 563.35516, found *m*/*z* = 563.3552.


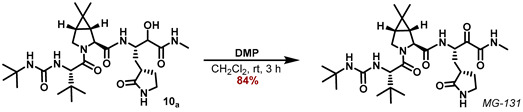


## Data Availability

Atomic coordinates and structure factors have been deposited in the Protein Data Bank (PDB), with the following PDB codes: SARS-CoV-2 M^pro^ complex with boceprevir, 7NBR; M^pro^ complex with telaprevir, 7NBS; M^pro^ complex with **MG-78**, 7QL8; M^pro^ complex with **MG-131**, 7Z0P; enterovirus A71 3C^pro^ complex with **MG-78**, 7QUB; Coxsackievirus B3 3C^pro^ complex with **MG-78**, 7QUW.

## Supplementary Materials

The following supporting information can be downloaded at: https://www.mdpi.com/article/10.3390/molecules27134292/s1: Figure S1: IC_50_ values for inhibition of SARS-CoV-2 M^pro^ by boceprevir, telaprevir, **MG-78**, and **MG-131**, as well as for inhibition of EV-A71 3C^pro^ and CVB3 3C^pro^ by **MG-78**; Table S1: Crystallographic data collection, structure solution, and refinement statistics; Figure S2: ^1^H and ^13^C NMR spectra of intermediates and final products.

## Sample Availability

Samples of the compounds are available from the authors.
