# Supplementary material for: From Repurposing to Redesign: Optimization of Boceprevir to Highly Potent Inhibitors of the SARS-CoV-2 Main Protease
Source: Molecules. 2022 Jul 4;27(13):4292. doi: 10.3390/molecules27134292 (PMC9268446; doi:10.3390/molecules27134292)
Supplement: Supplementary file 1 [file molecules-27-04292-s001.zip › molecules-1712233-supplementary.pdf]

Table S1. Diffraction data and model refinement statistics

Protein / Ligand	M^{pro} with boceprevir	M^{pro} with telaprevir	M^{pro} with MG-78	M^{pro} with MG-131	EV-A71-3C^{pro} with MG-78	CVB3-3C^{pro} with MG-78
**PDB entry**	7NBR	7NBS	7QL8	7Z0P	7QUB	7QUW

Data collection statistics

X-ray source	DESY P11	DESY P11	DESY P11	DESY P11	DESY P11	DESY P11
Wavelength [Å]	1.0332	1.0332	1.0332	1.0332	1.0332	1.0332
Vm [$Å^3$/Da]	2.02	2.10	2.27	2.32	2.32	2.22
Solvent content [%]	39.1	41.4	45.8	47.0	47.0	44.5
Space group	*C*2	*C*2	$P2_12_12$	*C*2	*P*432	*C*2

Unit cell dimensions [Å]	*a* = 113.77, *b* = 53.37, *c* = 45.95	*a* = 109.69, *b* = 54.63, *c* = 48.28	*a* = 45.52, *b* = 64.04, *c* = 105.38	a = 113.72, *b* = 51.67, *c* = 45.45	*a* = *b* = *c* = 122.34	*a* = 78.27, *b* = 64.29, *c* = 39.85
Unit cell dimensions [º]	α = γ = 90, β = 101.83	α = γ = 90, β = 101.40	α = β = γ = 90	α = γ = 90, β = 103.33	α = β = γ = 90	α = γ = 90, β = 115.52
Resolution range [a] [Å]	48.13 - 2.40 (2.53 - 2.40)	48.70 - 1.70 (1.79 - 1.70)	41.79 - 1.80 (1.90 - 1.80)	46.81 – 2.52 (2.62 - 2.52)	43.25 - 2.07 (2.19 - 2.07)	35.95 - 1.65 (1.74 - 1.65)
Number of observations	71,648 (10,224)	206,145 (28,922)	195,279 (30,344)	168,983 (25,182)	771,011 (123,429)	141,834 (20,568)
Number of unique reflections	10,684 (1,563)	30,908 (4,476)	29,378 (4,548)	49,867 (7,839)	19,792 (3,098)	43,026 (6,281)
Completeness [%]	99.9 (100.0)	100.0 (100.0)	99.4 (97.0)	99.0 (98.3)	99.9 (99.8)	98.1 (98.0)
Mean I/σ(I)	15.6 (1.7)	15.6 (1.8)	18.9 (3.5)	16.2 (1.0)	14.4 (1.0)	15.4 (1.9)
Multiplicity	6.7 (6.5)	6.7 (6.5)	5.6 (6.6)	3.5 (3.6)	38.9 (39.8)	6.7 (6.8)

R_{merge} [b] [%]	0.093 (1.179)	0.073 (1.089)	0.042 (0.480)	0.040 (0.167)	0.043 (0.282)	0.047 (0.797)
R_{pim} [c] [%]	0.039 (0.496)	0.030 (0.462)	0.025 (0.297)	0.039 (0.156)	0.010 (0.063)	0.020 (0.326)
$CC_{1/2}$ [d]	0.999 (0.663)	0.999 (0.688)	0.991 (0.693)	0.990 (0.392)	0.999 (0.522)	0.999 (0.874)
Wilson *B*-factor [Å^2]	48	23	44	53	46	51

Refinement statistics

R_{cryst} [e] /R_{free} [f] [%]	18.5/26.4	21.3/26.7	23.4/30.0	20.3/28.9	19.1/21.8	21.9/27.9
r.m.s.d. in bond lengths [Å]	0.008	0.009	0.007	0.007	0.012	0.007
r.m.s.d. in bond angles [°]	1.9	1.7	1.8	1.6	1.7	1.6
Clashscore [g]	4	5	3	8	5	4

Average *B*-factor for protein atoms [$Å^2$]	58	29	55	47	45	53
Average *B*-factor for ligand atoms [$Å^2$]	51	25	51	40	38	66
Average *B*-factor for water molecules [$Å^2$]	49	35	51	37	46	57
Number of protein atoms	2320	2352	2331	2347	1413	1393
Number of ligand atoms	37	49	39	40	39	39
Number of water molecules	48	155	75	37	52	51

Ramachandran plot

Preferred regions [%]	93	97	93	88	93	92
Allowed regions [%]	6	2	6	10	6	8
Outlier regions [%]	1	1	1	2	1	0

[a] The highest resolution shell is shown in parantheses.

[b] $R_{merge} = \sum_{hkl} \sum_{i=1}^{n} \left| I_i(hkl) - \bar{I}(hkl) \right| / \sum_{hkl} \sum_{i=1}^{n} I_i(hkl)$

[c] $R_{pim} = \sum_{hkl} \sqrt{1/(n-1)} \sum_{i=1}^{n} \left| I_i(hkl) - \bar{I}(hkl) \right| / \sum_{hkl} \sum_{i=1}^{n} I_i(hkl)$ [34]

[d] $CC_{1/2}$ is the correlation coefficient determined by two random half data sets [35]

[e] $R_{cryst} = \sum_{hkl} |F_o(hkl) - F_c(hkl)| / \sum_{hkl} |F_o(hkl)|$

[f] R_{free} was calculated for a test set of reflections (5%) omitted from the refinement.

[g] Clashscore is defined as the number of clashes calculated for the model per 1000 atoms (including hydrogens) of the model. Hydrogens were added by MolProbity [36]

Figure S1: Inhibitory activities of boceprevir-derived compounds vs. the SARS-CoV-2 M^{pro}

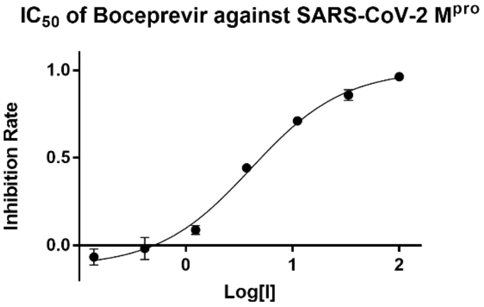

(a): IC_{50} = 4.1 ± 0.9 μM

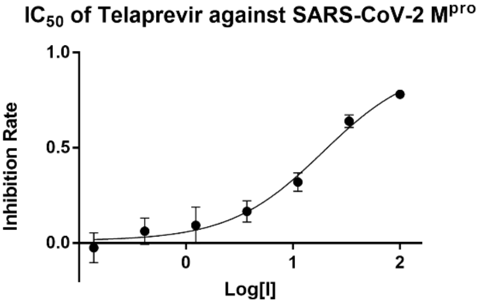

(b): IC_{50} = 19.0 ± 9.8 μM

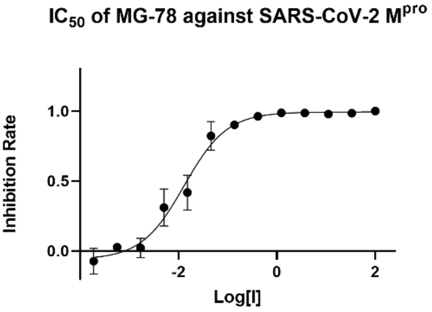

(c): IC_{50} = 13 ± 3 nM

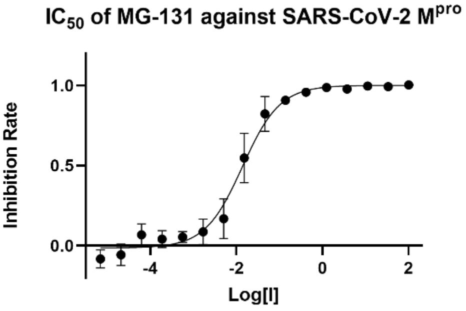

(d): IC_{50} = 14 ± 3 nM

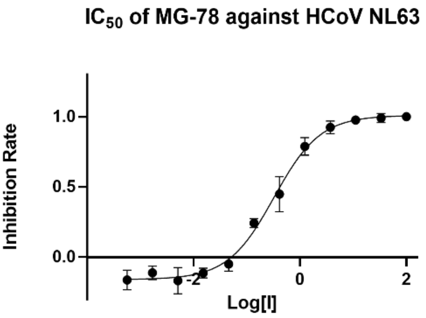

(e): IC_{50} = 0.33 ± 0.07 μM

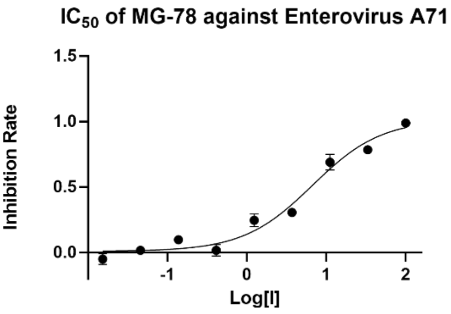

(f): IC_{50} = 6.7 ± 2.2 nM

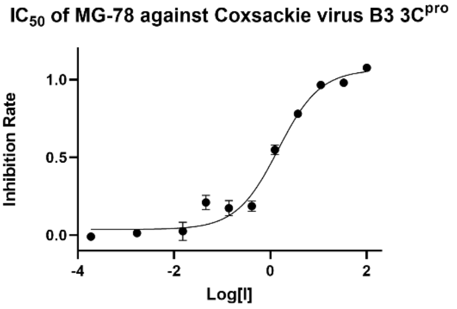

(g): IC_{50} = 1.45 ± 0.40 μM

Figure S1. Inhibitory activities against the SARS-CoV-2 M^{pro} of boceprevir (a), telaprevir (b), MG-78 (c), and MG-131 (d); inhibitory activities of compound MG-78 against HCoV-NL63 M^{pro} (e), EV-A71 $3C^{pro}$ (f), and CVB3 $3C^{pro}$ (g). IC_{50} values have been derived from FRET-based enzymatic assays described in the main text.

Figure S2. NMR spectra

NMR spectra of MG-78 and its intermediates

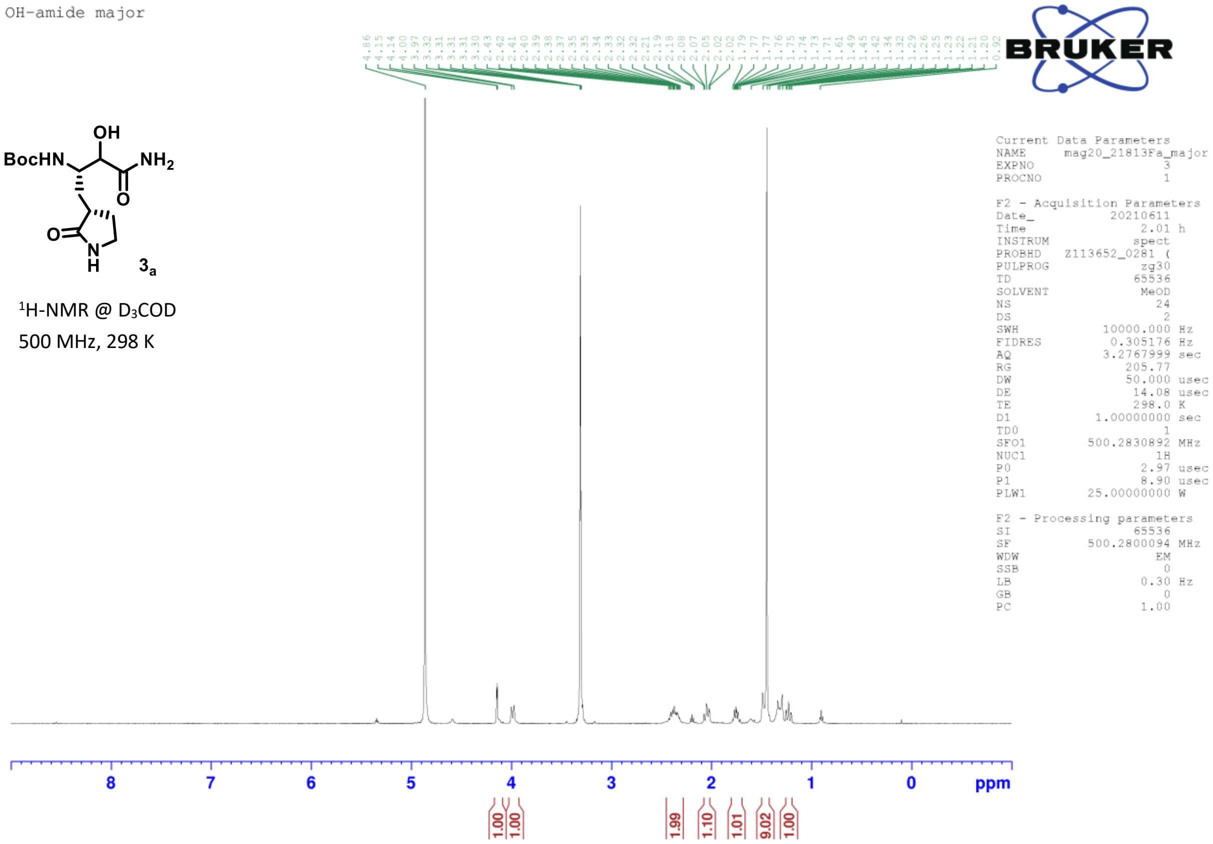

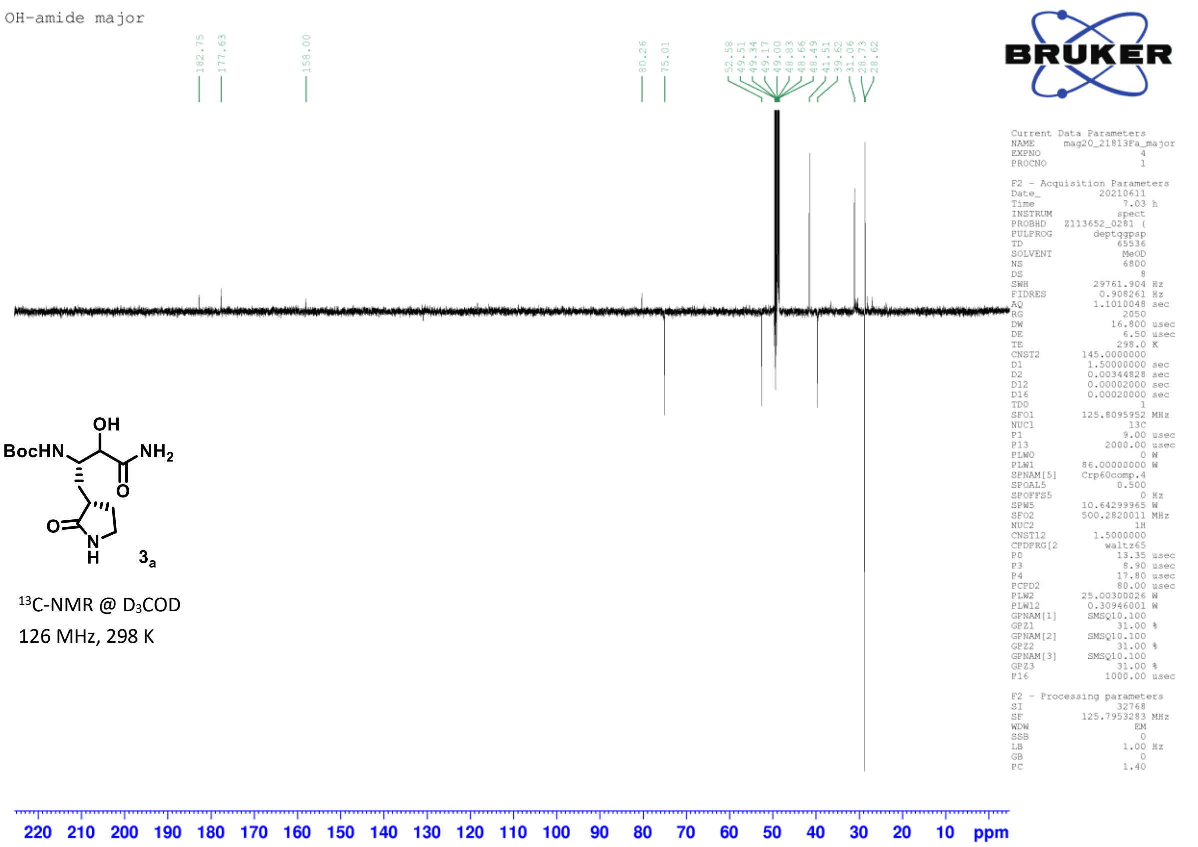
OH-amide major
182.75
177.63
158.00
80.26
75.01
52.58
49.51
49.34
49.17
49.00
48.83
48.66
48.49
41.51
39.62
31.06
28.73
28.62
BocHN
OH
NH2
O
O
N
H
3a
13C-NMR @ D3COD
126 MHz, 298 K
BRUKER
Current Data Parameters
NAME mag20_21813Fa_major
EXPNO 4
PROCNO 1
F2 - Acquisition Parameters
Date_ 20210611
Time 7.03 h
INSTRUM spect
PROBHD Z113652_0281 (
PULPROG deptqgpsp
TD 65536
SOLVENT MeOD
NS 6800
DS 8
SWH 29761.904 Hz
FIDRES 0.908261 Hz
AQ 1.1010048 sec
RG 2050
DW 16.800 usec
DE 6.50 usec
TE 298.0 K
CNST2 145.0000000
D1 1.50000000 sec
D2 0.00344828 sec
D12 0.00002000 sec
D16 0.00020000 sec
TD0 1
SFO1 125.8095952 MHz
NUC1 13C
P1 9.00 usec
P13 2000.00 usec
PLW0 0 W
PLW1 86.00000000 W
SPNAM[5] Crp60comp.4
SPOAL5 0.500
SPOFFS5 0 Hz
SPW5 10.64299965 W
SFO2 500.2820011 MHz
NUC2 1H
CNST12 1.5000000
CPDPRG[2 waltz65
P0 13.35 usec
P3 8.90 usec
P4 17.80 usec
PCPD2 80.00 usec
PLW2 25.00300026 W
PLW12 0.30946001 W
GPNAM[1] SMSQ10.100
GPZ1 31.00 %
GPNAM[2] SMSQ10.100
GPZ2 31.00 %
GPNAM[3] SMSQ10.100
GPZ3 31.00 %
P16 1000.00 usec
F2 - Processing parameters
SI 32768
SF 125.7953283 MHz
WDW EM
SSB 0
LB 1.00 Hz
GB 0
PC 1.40
220 210 200 190 180 170 160 150 140 130 120 110 100 90 80 70 60 50 40 30 20 10 ppm

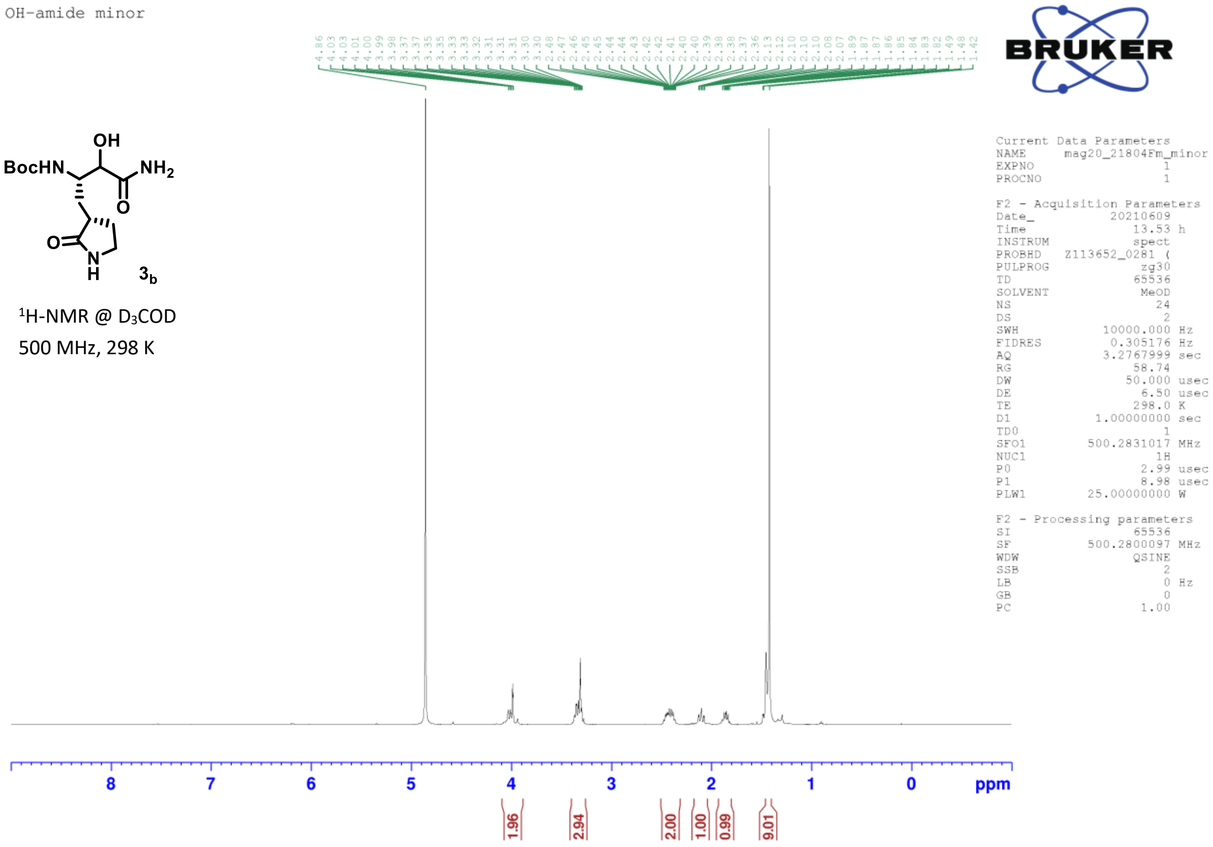
OH-amide minor
BocHN
OH
NH2
O
O
N
H
3b
1H-NMR @ D3COD
500 MHz, 298 K
BRUKER
Current Data Parameters
NAME mag20_21804Fm_minor
EXPNO 1
PROCNO 1
F2 - Acquisition Parameters
Date_ 20210609
Time 13.53 h
INSTRUM spect
PROBHD Z113652_0281 (
PULPROG zg30
TD 65536
SOLVENT MeOD
NS 24
DS 2
SWH 10000.000 Hz
FIDRES 0.305176 Hz
AQ 3.2767999 sec
RG 58.74
DW 50.000 usec
DE 6.50 usec
TE 298.0 K
D1 1.00000000 sec
TD0 1
SFO1 500.2831017 MHz
NUC1 1H
P0 2.99 usec
P1 8.98 usec
PLW1 25.00000000 W
F2 - Processing parameters
SI 65536
SF 500.2800097 MHz
WDW QSINE
SSB 2
LB 0 Hz
GB 0
PC 1.00
8 7 6 5 4 3 2 1 0 ppm
1.96
2.94
2.00
1.00
0.99
9.01

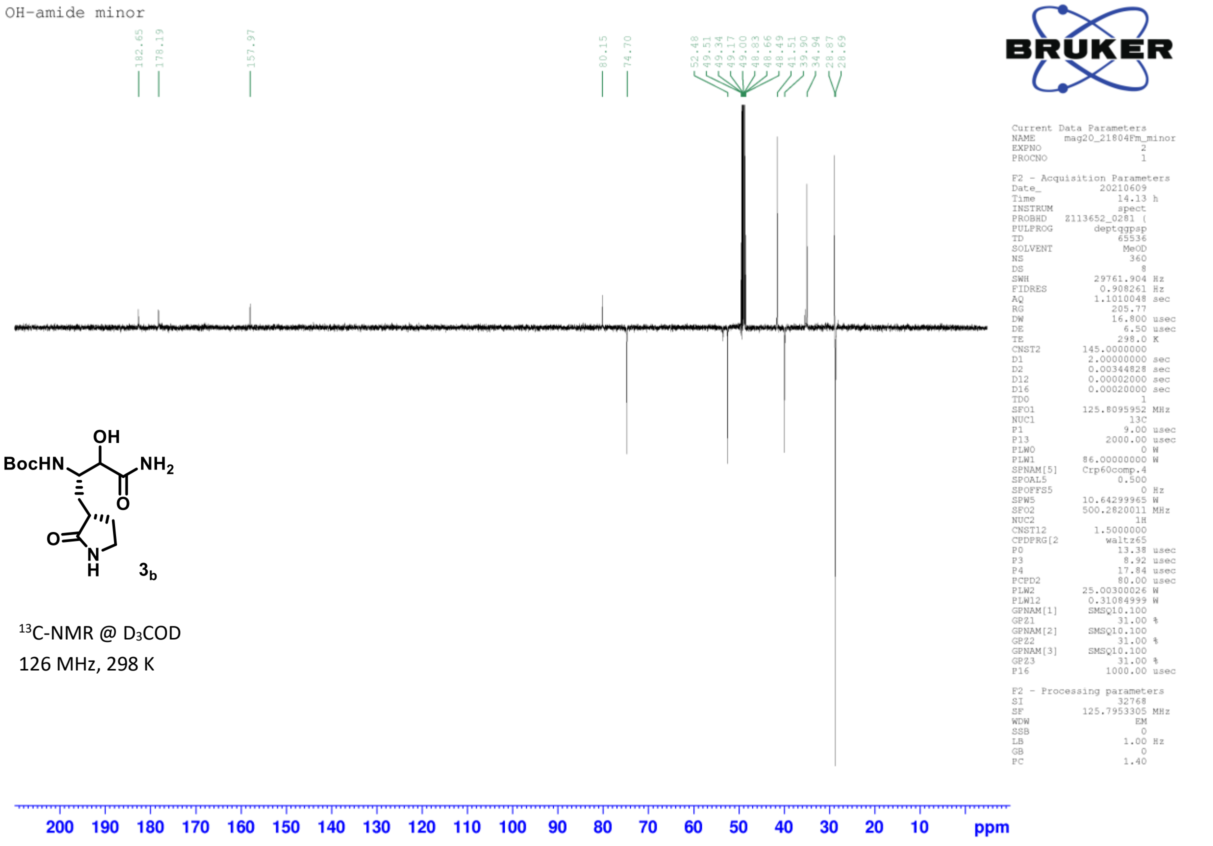
OH-amide minor
182.65
178.19
157.97
80.15
74.70
52.48
49.51
49.34
49.17
49.00
48.83
48.66
48.49
41.51
39.90
34.94
28.87
28.69
BocHN
OH
NH2
O
O
N
H
3b
13C-NMR @ D3COD
126 MHz, 298 K
BRUKER
Current Data Parameters
NAME mag20_21804Fm_minor
EXPNO 2
PROCNO 1
F2 - Acquisition Parameters
Date_ 20210609
Time 14.13 h
INSTRUM spect
PROBHD Z113652_0281 (
PULPROG deptqgpsp
TD 65536
SOLVENT MeOD
NS 360
DS 8
SWH 29761.904 Hz
FIDRES 0.908261 Hz
AQ 1.1010048 sec
RG 205.77
DW 16.800 usec
DE 6.50 usec
TE 298.0 K
CNST2 145.0000000
D1 2.00000000 sec
D2 0.00344828 sec
D12 0.00002000 sec
D16 0.00020000 sec
TD0 1
SFO1 125.8095952 MHz
NUC1 13C
P1 9.00 usec
P13 2000.00 usec
PLW0 0 W
PLW1 86.00000000 W
SPNAM[5] Crp60comp.4
SPOAL5 0.500
SPOFFS5 0 Hz
SPW5 10.64299965 W
SFO2 500.2820011 MHz
NUC2 1H
CNST12 1.5000000
CPDPRG[2 waltz65
P0 13.38 usec
P3 8.92 usec
P4 17.84 usec
PCPD2 80.00 usec
PLW2 25.00300026 W
PLW12 0.31084999 W
GPNAM[1] SMSQ10.100
GPZ1 31.00 %
GPNAM[2] SMSQ10.100
GPZ2 31.00 %
GPNAM[3] SMSQ10.100
GPZ3 31.00 %
P16 1000.00 usec
F2 - Processing parameters
SI 32768
SF 125.7953305 MHz
WDW EM
SSB 0
LB 1.00 Hz
GB 0
PC 1.40
200 190 180 170 160 150 140 130 120 110 100 90 80 70 60 50 40 30 20 10 ppm

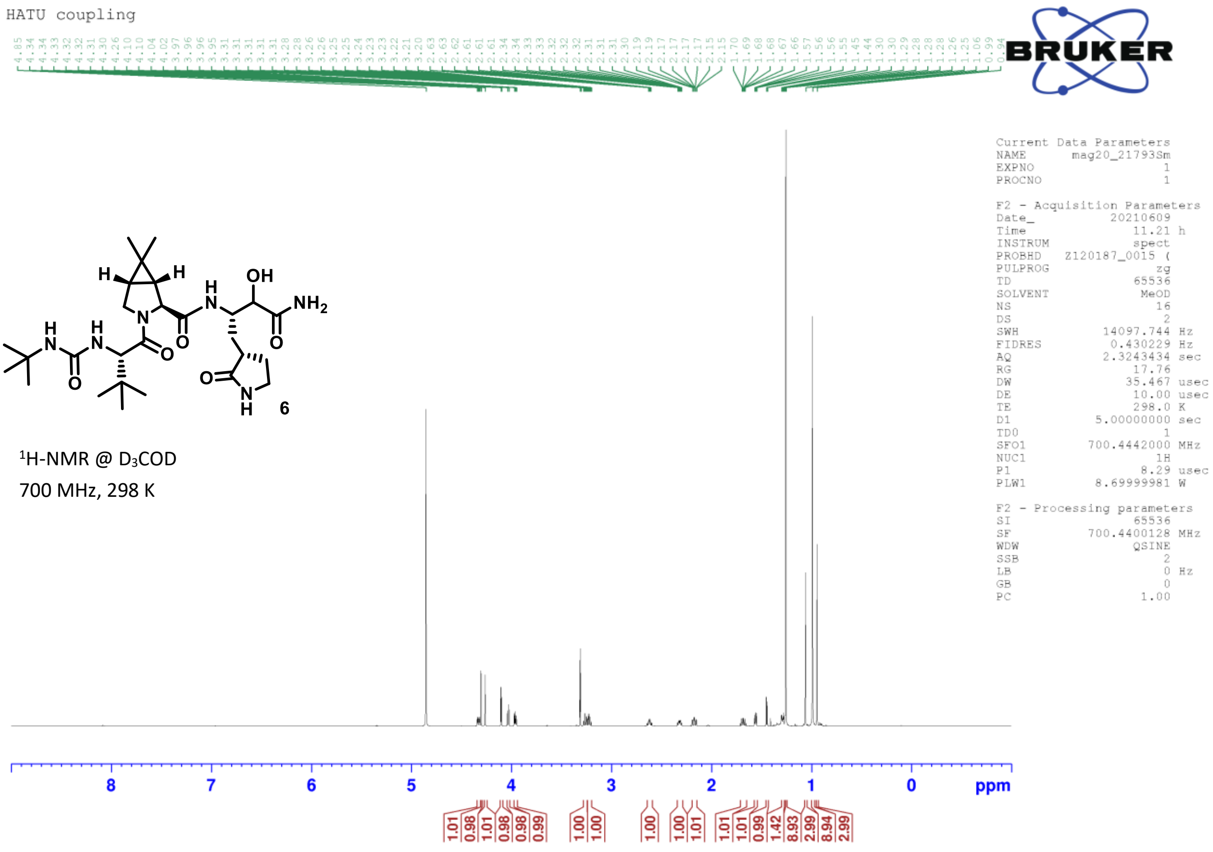
HATU coupling
H
H
OH
H
N
NH2
H
N
H
N
N
O
O
O
O
O
N
H
6
1H-NMR @ D3COD
700 MHz, 298 K
BRUKER
Current Data Parameters
NAME mag20_21793Sm
EXPNO 1
PROCNO 1
F2 - Acquisition Parameters
Date_ 20210609
Time 11.21 h
INSTRUM spect
PROBHD Z120187_0015 (
PULPROG zg
TD 65536
SOLVENT MeOD
NS 16
DS 2
SWH 14097.744 Hz
FIDRES 0.430229 Hz
AQ 2.3243434 sec
RG 17.76
DW 35.467 usec
DE 10.00 usec
TE 298.0 K
D1 5.00000000 sec
TD0 1
SFO1 700.4442000 MHz
NUC1 1H
P1 8.29 usec
PLW1 8.69999981 W
F2 - Processing parameters
SI 65536
SF 700.4400128 MHz
WDW QSINE
SSB 2
LB 0 Hz
GB 0
PC 1.00
8 7 6 5 4 3 2 1 0 ppm
1.01
0.98
1.01
0.98
0.99
0.99
1.00
1.00
1.00
1.00
1.01
1.01
0.99
1.42
8.93
2.99
8.94
2.99

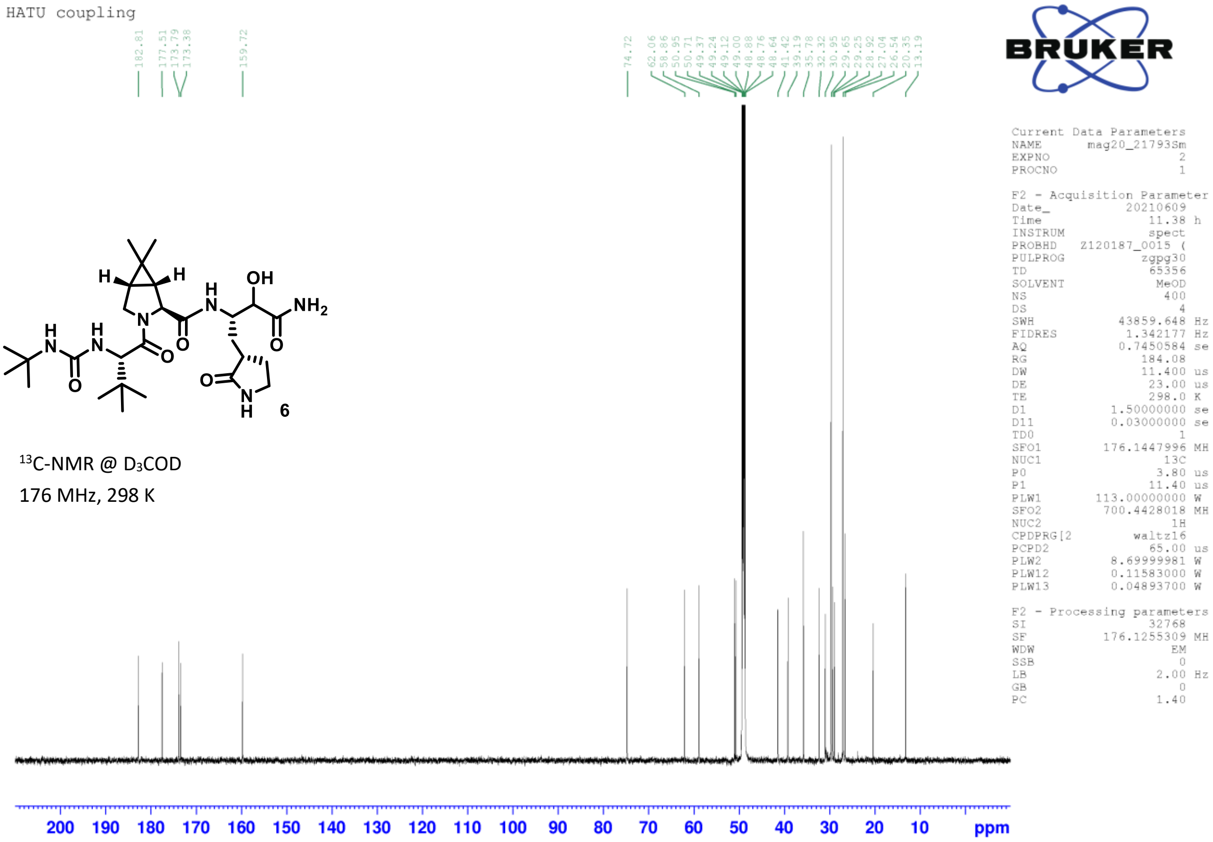
HATU coupling
182.81
177.51
173.79
173.38
159.72
74.72
62.06
58.86
50.95
50.71
49.37
49.24
49.12
49.00
48.88
48.76
48.64
41.42
39.19
35.78
32.32
30.95
29.65
29.25
28.92
27.04
26.54
20.35
13.19
BRUKER
6
13C-NMR @ D3COD
176 MHz, 298 K
Current Data Parameters
NAME mag20_21793Sm
EXPNO 2
PROCNO 1
F2 - Acquisition Parameter
Date_ 20210609
Time 11.38 h
INSTRUM spect
PROBHD Z120187_0015 (
PULPROG zgpg30
TD 65356
SOLVENT MeOD
NS 400
DS 4
SWH 43859.648 Hz
FIDRES 1.342177 Hz
AQ 0.7450584 se
RG 184.08
DW 11.400 us
DE 23.00 us
TE 298.0 K
D1 1.50000000 se
D11 0.03000000 se
TD0 1
SFO1 176.1447996 MH
NUC1 13C
P0 3.80 us
P1 11.40 us
PLW1 113.00000000 W
SFO2 700.4428018 MH
NUC2 1H
CPDPRG[2 waltz16
PCPD2 65.00 us
PLW2 8.69999981 W
PLW12 0.11583000 W
PLW13 0.04893700 W
F2 - Processing parameters
SI 32768
SF 176.1255309 MH
WDW EM
SSB 0
LB 2.00 Hz
GB 0
PC 1.40
200 190 180 170 160 150 140 130 120 110 100 90 80 70 60 50 40 30 20 10 ppm

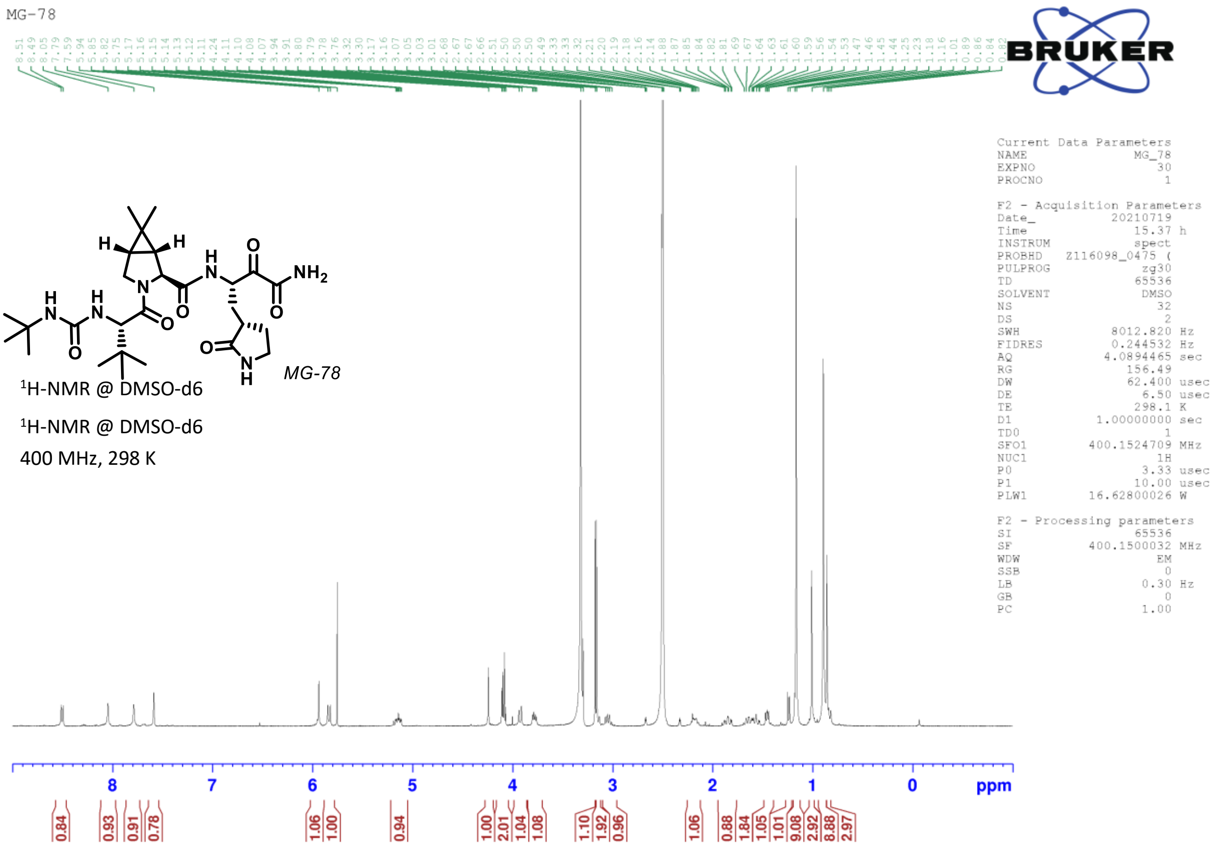
MG-78
BRUKER
MG-78
1H-NMR @ DMSO-d6
1H-NMR @ DMSO-d6
400 MHz, 298 K
Current Data Parameters
NAME MG_78
EXPNO 30
PROCNO 1
F2 - Acquisition Parameters
Date_ 20210719
Time 15.37 h
INSTRUM spect
PROBHD Z116098_0475 (
PULPROG zg30
TD 65536
SOLVENT DMSO
NS 32
DS 2
SWH 8012.820 Hz
FIDRES 0.244532 Hz
AQ 4.0894465 sec
RG 156.49
DW 62.400 usec
DE 6.50 usec
TE 298.1 K
D1 1.00000000 sec
TD0 1
SFO1 400.1524709 MHz
NUC1 1H
P0 3.33 usec
P1 10.00 usec
PLW1 16.62800026 W
F2 - Processing parameters
SI 65536
SF 400.1500032 MHz
WDW EM
SSB 0
LB 0.30 Hz
GB 0
PC 1.00
8 7 6 5 4 3 2 1 0 ppm
0.84
0.93
0.91
0.78
1.06
1.00
0.94
1.00
2.01
1.04
1.08
1.10
1.92
0.96
1.06
0.88
1.84
1.05
1.01
9.08
2.92
8.88
2.97

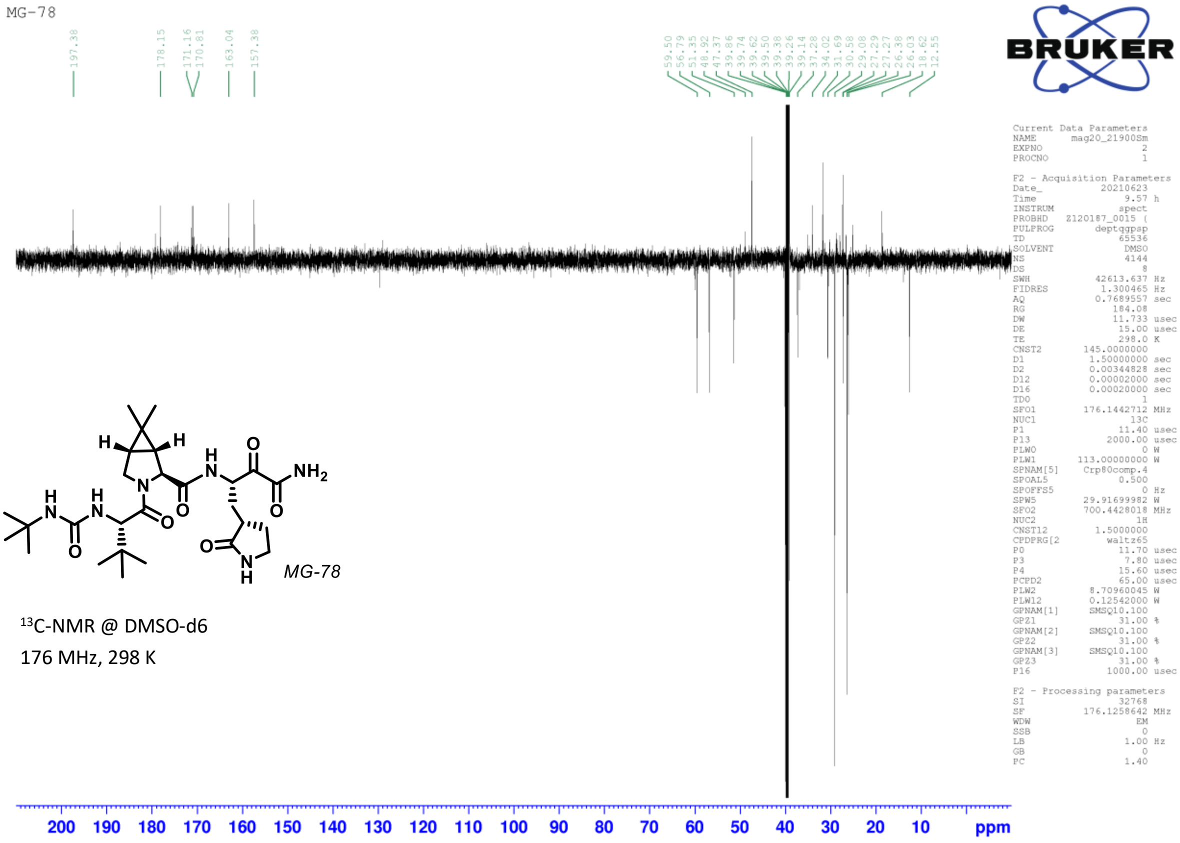
MG-78
197.38
178.15
171.16
170.81
163.04
157.38
59.50
56.79
51.35
48.92
47.37
39.86
39.74
39.62
39.50
39.38
39.26
39.14
37.28
34.02
31.69
31.58
29.08
27.29
27.27
26.38
26.03
18.62
12.55
BRUKER
Current Data Parameters
NAME mag20_21900Sm
EXPNO 2
PROCNO 1
F2 - Acquisition Parameters
Date_ 20210623
Time 9.57 h
INSTRUM spect
PROBHD Z120187_0015 (
PULPROG deptqgpsp
TD 65536
SOLVENT DMSO
NS 4144
DS 8
SWH 42613.637 Hz
FIDRES 1.300465 Hz
AQ 0.7689557 sec
RG 184.08
DW 11.733 usec
DE 15.00 usec
TE 298.0 K
CNST2 145.0000000
D1 1.50000000 sec
D2 0.00344828 sec
D12 0.00002000 sec
D16 0.00020000 sec
TD0 1
SFO1 176.1442712 MHz
NUC1 13C
P1 11.40 usec
P13 2000.00 usec
PLW0 0 W
PLW1 113.00000000 W
SPNAM[5] Crp80comp.4
SPOAL5 0.500
SPOFFS5 0 Hz
SPW5 29.91699982 W
SFO2 700.4428018 MHz
NUC2 1H
CNST12 1.5000000
CPDPRG[2 waltz65
P0 11.70 usec
P3 7.80 usec
P4 15.60 usec
PCPD2 65.00 usec
PLW2 8.70960045 W
PLW12 0.12542000 W
GPNAM[1] SMSQ10.100
GPZ1 31.00 %
GPNAM[2] SMSQ10.100
GPZ2 31.00 %
GPNAM[3] SMSQ10.100
GPZ3 31.00 %
P16 1000.00 usec
F2 - Processing parameters
SI 32768
SF 176.1258642 MHz
WDW EM
SSB 0
LB 1.00 Hz
GB 0
PC 1.40
MG-78
13C-NMR @ DMSO-d6
176 MHz, 298 K
200 190 180 170 160 150 140 130 120 110 100 90 80 70 60 50 40 30 20 10 ppm

NMR spectra of MG-131 and its intermediates

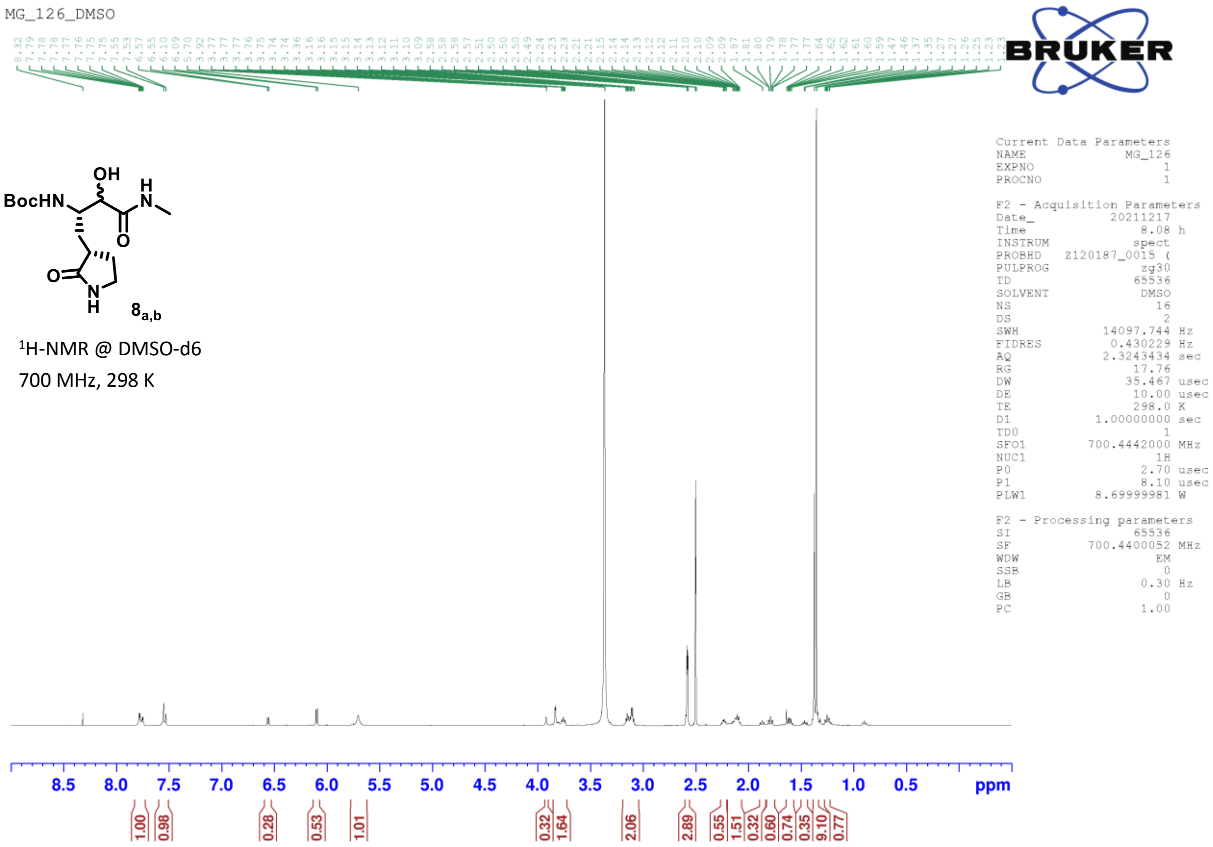

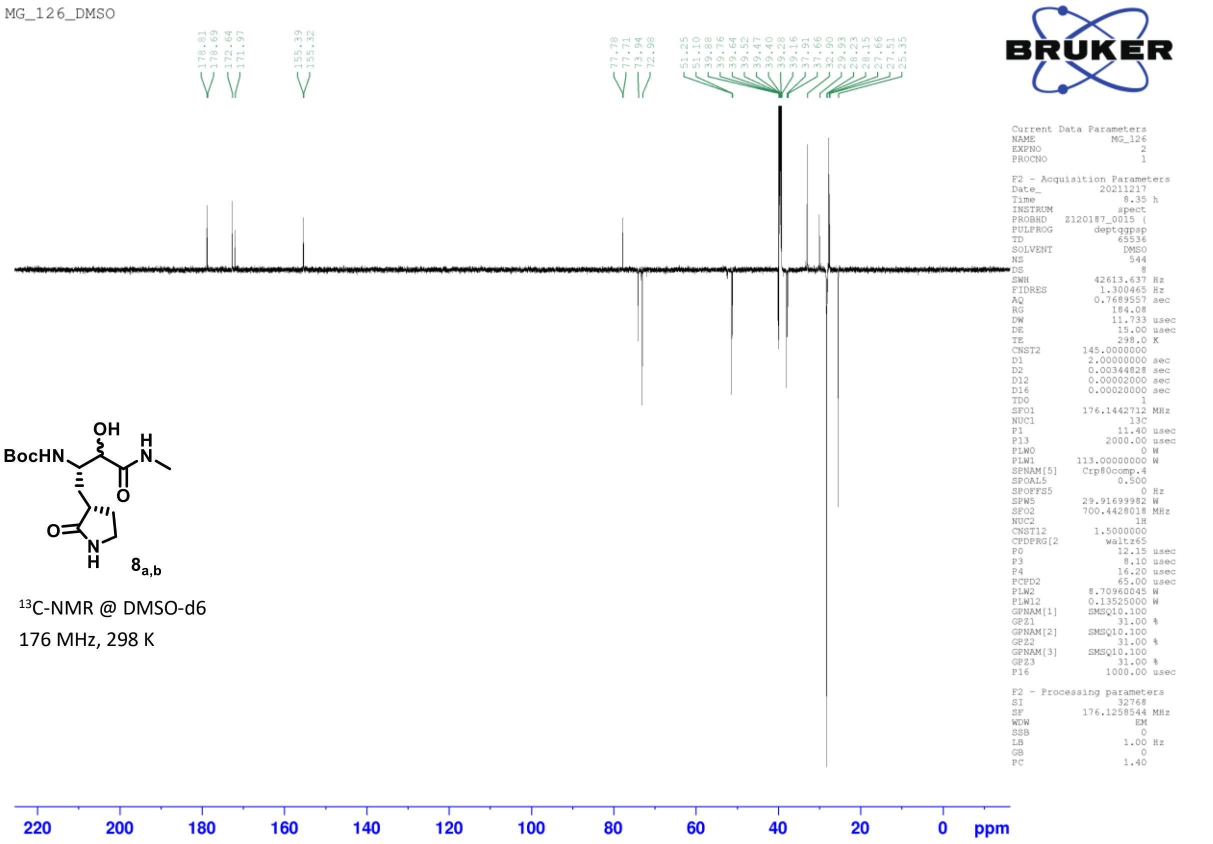

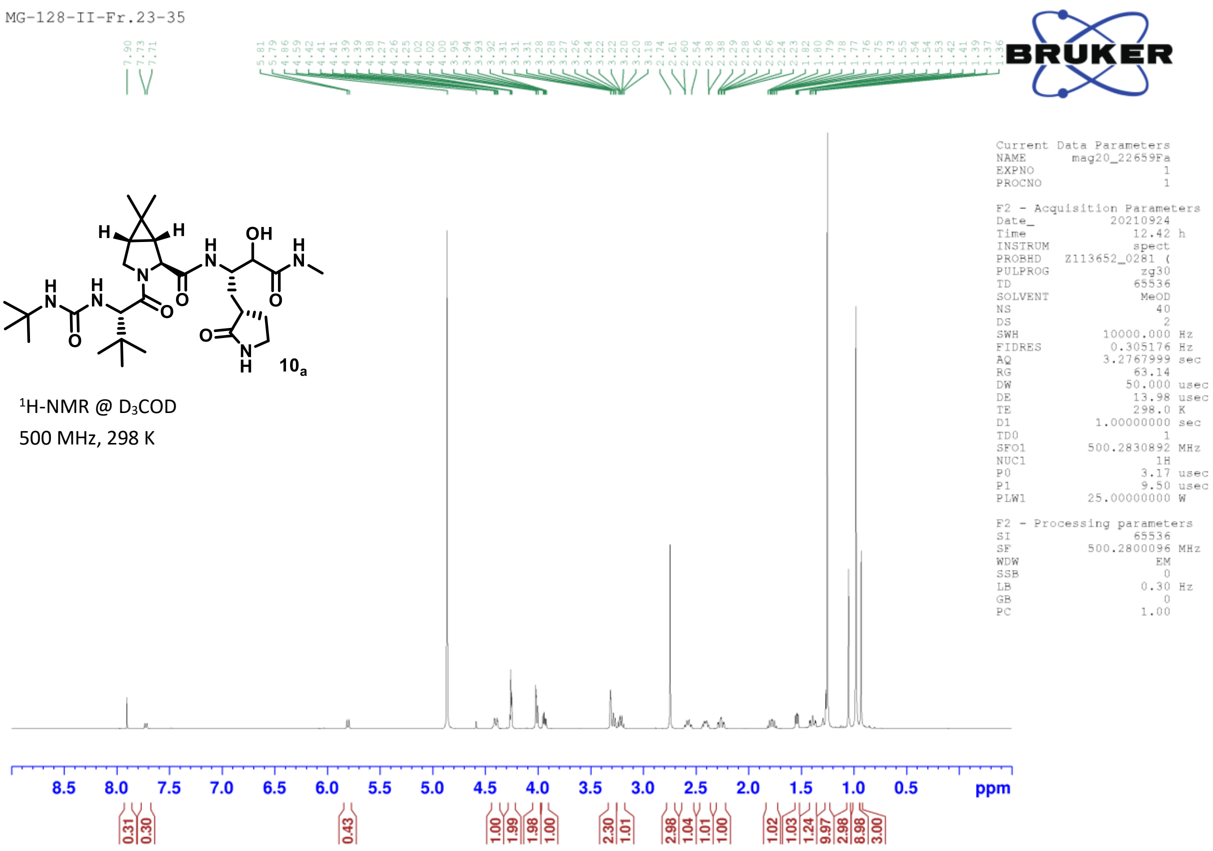
MG-128-II-Fr.23-35
7.90 7.73 7.71
5.81 5.79 4.86 4.59 4.42 4.41 4.41 4.39 4.39 4.38 4.27 4.26 4.25 4.02 4.02 4.00 3.95 3.94 3.93 3.92 3.31 3.31 3.31 3.28 3.28 3.27 3.26 3.24 3.22 3.22 3.20 3.20 3.18 2.74 2.61 2.60 2.54 2.38 2.38 2.29 2.28 2.26 2.26 2.24 2.23 1.82 1.80 1.79 1.78 1.77 1.76 1.75 1.73 1.55 1.54 1.54 1.53 1.42 1.41 1.39 1.37 1.36
H
H
H
N
H
N
OH
H
N
N
O
O
O
O
O
N
H
10a
^{1}H-NMR @ D$_3$COD
500 MHz, 298 K
Current Data Parameters
NAME mag20_22659Fa
EXPNO 1
PROCNO 1
F2 - Acquisition Parameters
Date_ 20210924
Time 12.42 h
INSTRUM spect
PROBHD Z113652_0281 (
PULPROG zg30
TD 65536
SOLVENT MeOD
NS 40
DS 2
SWH 10000.000 Hz
FIDRES 0.305176 Hz
AQ 3.2767999 sec
RG 63.14
DW 50.000 usec
DE 13.98 usec
TE 298.0 K
D1 1.00000000 sec
TD0 1
SFO1 500.2830892 MHz
NUC1 1H
P0 3.17 usec
P1 9.50 usec
PLW1 25.00000000 W
F2 - Processing parameters
SI 65536
SF 500.2800096 MHz
WDW EM
SSB 0
LB 0.30 Hz
GB 0
PC 1.00
8.5 8.0 7.5 7.0 6.5 6.0 5.5 5.0 4.5 4.0 3.5 3.0 2.5 2.0 1.5 1.0 0.5 ppm
0.31 0.30 0.43 1.00 1.99 1.98 1.00 2.30 1.01 2.98 1.04 1.01 1.00 1.02 1.03 1.24 9.97 2.98 8.98 3.00

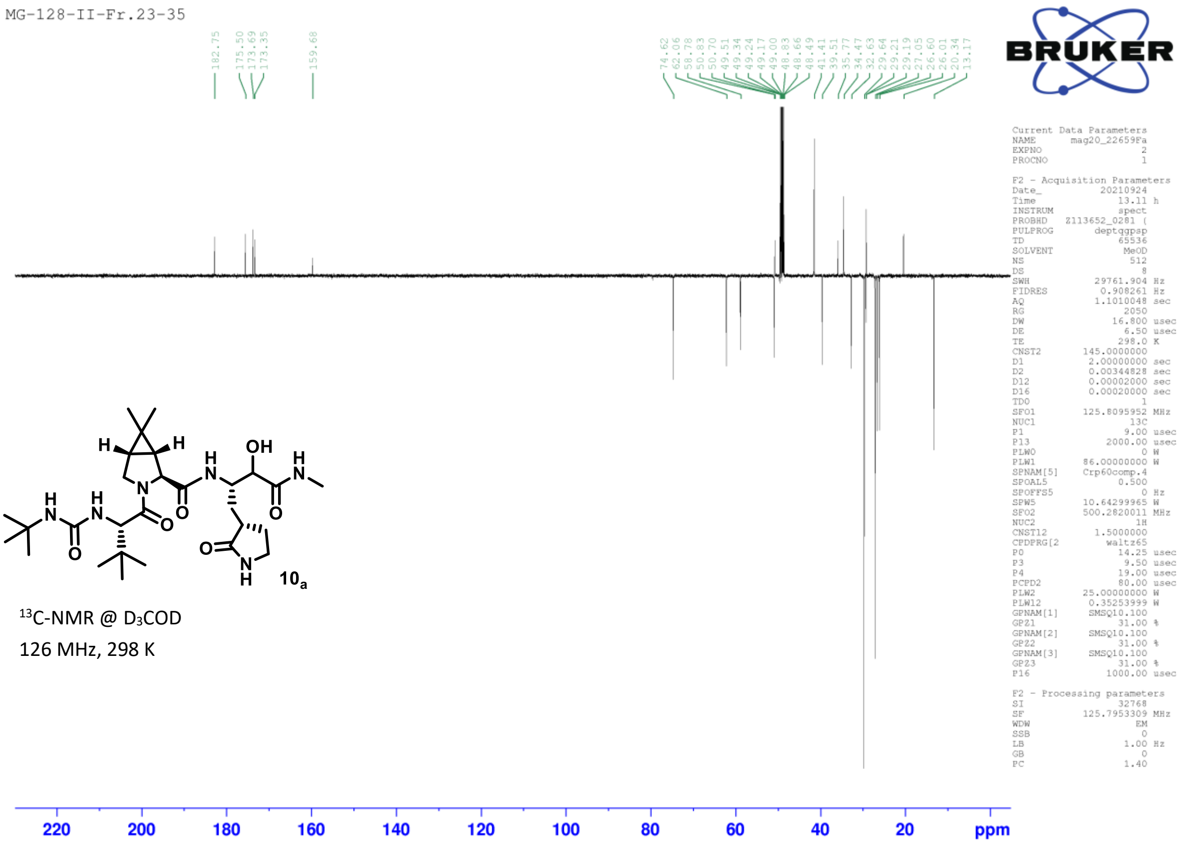
MG-128-II-Fr.23-35
182.75 175.50 173.69 173.35 159.68
74.62 62.06 58.78 50.83 50.70 49.51 49.34 49.24 49.17 49.00 48.83 48.66 48.49 41.41 39.51 35.77 34.47 32.63 29.64 29.21 29.19 27.05 26.60 26.01 20.34 13.17
H
H
H
N
H
N
OH
H
N
N
O
O
O
O
O
N
H
10a
^{13}C-NMR @ D$_3$COD
126 MHz, 298 K
Current Data Parameters
NAME mag20_22659Fa
EXPNO 2
PROCNO 1
F2 - Acquisition Parameters
Date_ 20210924
Time 13.11 h
INSTRUM spect
PROBHD Z113652_0281 (
PULPROG deptqgpsp
TD 65536
SOLVENT MeOD
NS 512
DS 8
SWH 29761.904 Hz
FIDRES 0.908261 Hz
AQ 1.1010048 sec
RG 2050
DW 16.800 usec
DE 6.50 usec
TE 298.0 K
CNST2 145.0000000
D1 2.00000000 sec
D2 0.00344828 sec
D12 0.00002000 sec
D16 0.00020000 sec
TD0 1
SFO1 125.8095952 MHz
NUC1 13C
P1 9.00 usec
P13 2000.00 usec
PLW0 0 W
PLW1 86.00000000 W
SPNAM[5] Crp60comp.4
SPOAL5 0.500
SPOFFS5 0 Hz
SPW5 10.64299965 W
SFO2 500.2820011 MHz
NUC2 1H
CNST12 1.5000000
CPDPRG[2 waltz65
P0 14.25 usec
P3 9.50 usec
P4 19.00 usec
PCPD2 80.00 usec
PLW2 25.00000000 W
PLW12 0.35253999 W
GPNAM[1] SMSQ10.100
GPZ1 31.00 %
GPNAM[2] SMSQ10.100
GPZ2 31.00 %
GPNAM[3] SMSQ10.100
GPZ3 31.00 %
P16 1000.00 usec
F2 - Processing parameters
SI 32768
SF 125.7953309 MHz
WDW EM
SSB 0
LB 1.00 Hz
GB 0
PC 1.40
220 200 180 160 140 120 100 80 60 40 20 ppm

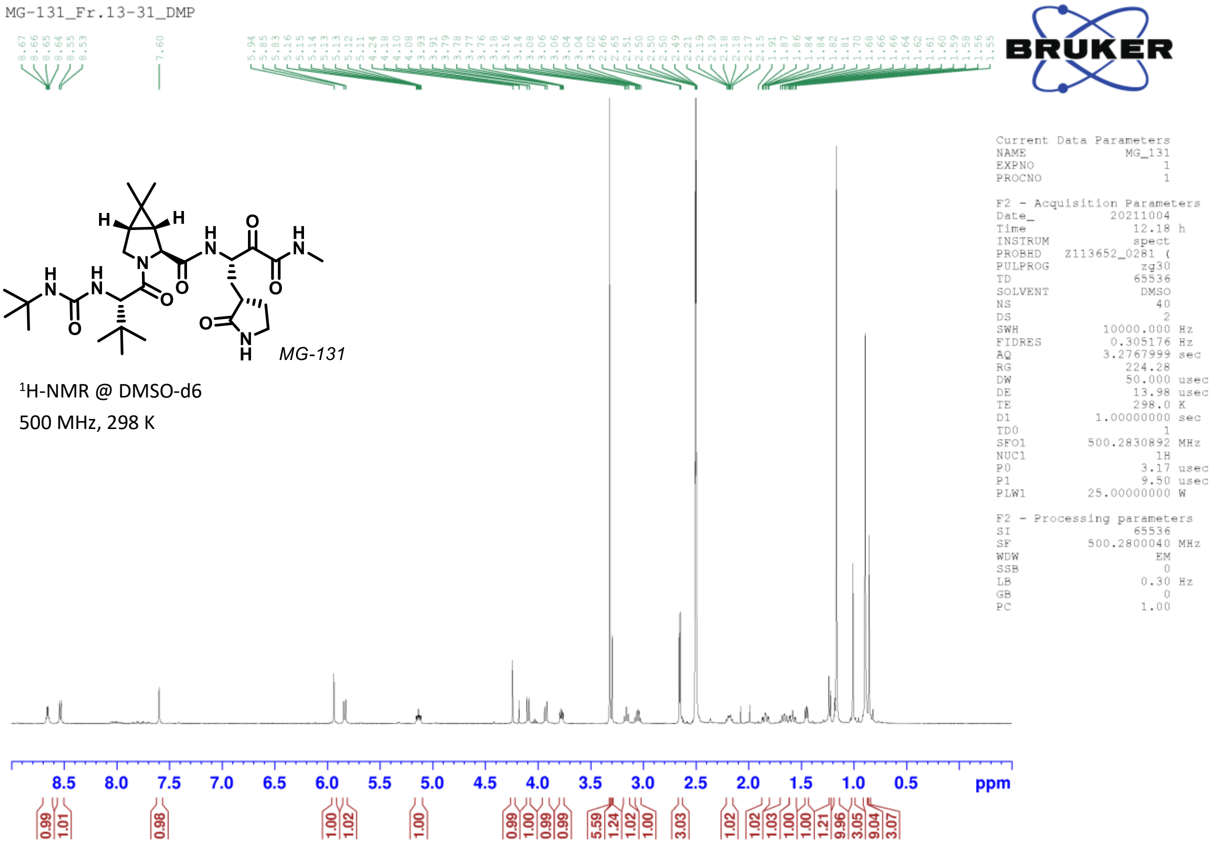
MG-131_Fr.13-31_DMP
8.67 8.66 8.65 8.64 8.55 8.53 7.60 5.94 5.85 5.83 5.16 5.15 5.14 5.13 5.13 5.12 5.11 4.24 4.18 4.10 4.08 3.93 3.91 3.79 3.78 3.77 3.76 3.18 3.16 3.14 3.08 3.06 3.04 3.02 2.66 2.65 2.51 2.50 2.50 2.50 2.49 2.21 2.19 2.19 2.18 2.18 2.17 2.15 1.91 1.87 1.86 1.84 1.84 1.82 1.70 1.68 1.66 1.64 1.62 1.61 1.60 1.59 1.58 1.56 1.55
BRUKER
Current Data Parameters
NAME MG_131
EXPNO 1
PROCNO 1
F2 - Acquisition Parameters
Date_ 20211004
Time 12.18 h
INSTRUM spect
PROBHD Z113652_0281 (
PULPROG zg30
TD 65536
SOLVENT DMSO
NS 40
DS 2
SWH 10000.000 Hz
FIDRES 0.305176 Hz
AQ 3.2767999 sec
RG 224.28
DW 50.000 usec
DE 13.98 usec
TE 298.0 K
D1 1.00000000 sec
TD0 1
SFO1 500.2830892 MHz
NUC1 1H
P0 3.17 usec
P1 9.50 usec
PLW1 25.00000000 W
F2 - Processing parameters
SI 65536
SF 500.2800040 MHz
WDW EM
SSB 0
LB 0.30 Hz
GB 0
PC 1.00
MG-131
1H-NMR @ DMSO-d6
500 MHz, 298 K
8.5 8.0 7.5 7.0 6.5 6.0 5.5 5.0 4.5 4.0 3.5 3.0 2.5 2.0 1.5 1.0 0.5 ppm
0.99 1.01 0.98 1.00 1.02 1.00 0.99 1.00 0.99 0.99 5.59 1.24 1.02 1.00 3.03 1.02 1.02 1.03 1.00 1.00 1.21 9.96 3.05 9.04 3.07

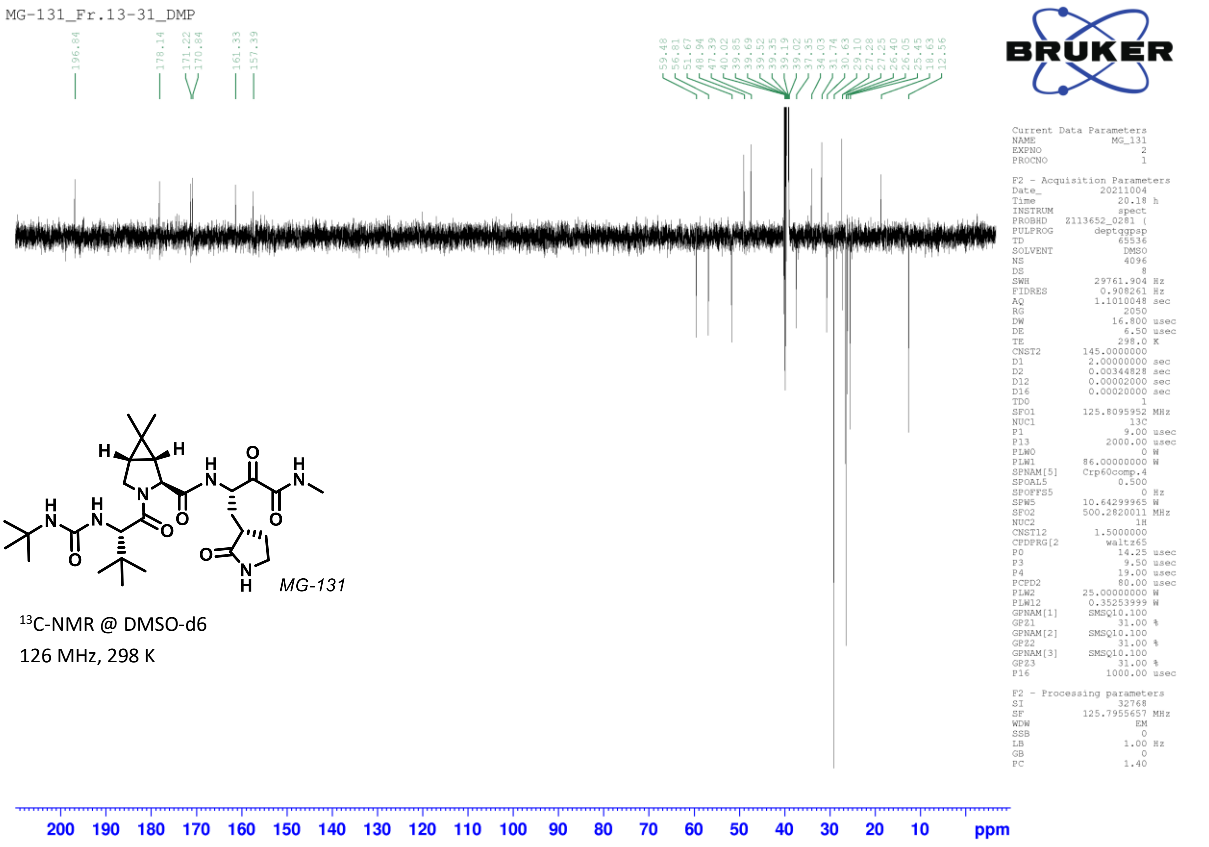
MG-131_Fr.13-31_DMP
196.84 178.14 171.22 170.84 161.33 157.39 59.48 56.81 51.67 47.94 47.39 40.02 39.85 39.69 39.52 39.35 39.19 39.02 37.35 34.03 31.74 30.63 29.10 27.28 27.25 26.40 26.05 25.45 18.63 12.56
BRUKER
Current Data Parameters
NAME MG_131
EXPNO 2
PROCNO 1
F2 - Acquisition Parameters
Date_ 20211004
Time 20.18 h
INSTRUM spect
PROBHD Z113652_0281 (
PULPROG deptqgpsp
TD 65536
SOLVENT DMSO
NS 4096
DS 8
SWH 29761.904 Hz
FIDRES 0.908261 Hz
AQ 1.1010048 sec
RG 2050
DW 16.800 usec
DE 6.50 usec
TE 298.0 K
CNST2 145.0000000
D1 2.00000000 sec
D2 0.00344828 sec
D12 0.00002000 sec
D16 0.00020000 sec
TD0 1
SFO1 125.8095952 MHz
NUC1 13C
P1 9.00 usec
P13 2000.00 usec
PLW0 0 W
PLW1 86.00000000 W
SPNAM[5] Crp60comp.4
SPOAL5 0.500
SPOFFS5 0 Hz
SPW5 10.64299965 W
SFO2 500.2820011 MHz
NUC2 1H
CNST12 1.5000000
CPDPRG[2 waltz65
P0 14.25 usec
P3 9.50 usec
P4 19.00 usec
PCPD2 80.00 usec
PLW2 25.00000000 W
PLW12 0.35253999 W
GPNAM[1] SMSQ10.100
GPZ1 31.00 %
GPNAM[2] SMSQ10.100
GPZ2 31.00 %
GPNAM[3] SMSQ10.100
GPZ3 31.00 %
P16 1000.00 usec
F2 - Processing parameters
SI 32768
SF 125.7955657 MHz
WDW EM
SSB 0
LB 1.00 Hz
GB 0
PC 1.40
MG-131
13C-NMR @ DMSO-d6
126 MHz, 298 K
200 190 180 170 160 150 140 130 120 110 100 90 80 70 60 50 40 30 20 10 ppm
